# FEM–BEM coupling for the large-body limit in micromagnetics

**DOI:** 10.1016/j.cam.2014.11.042

**Published:** 2015-06

**Authors:** M. Aurada, J.M. Melenk, D. Praetorius

**Affiliations:** Institute for Analysis and Scientific Computing, Vienna University of Technology, Wiedner Hauptstraße 8-10, A-1040 Wien, Austria

**Keywords:** Computational micromagnetism, FEM–BEM coupling

## Abstract

We present and analyze a coupled finite element–boundary element method for a model in stationary micromagnetics. The finite element part is based on mixed conforming elements. For two- and three-dimensional settings, we show well-posedness of the discrete problem and present an *a priori* error analysis for the case of lowest order elements.

## 1 Introduction

Stationary micromagnetism is a theory that is successfully used to describe and predict magnetic phenomena, focusing typically on effects on a macroscopic length scale. The various models currently in use originate from a classical approach by Landau and Lifshitz,  [1], where the magnetization state $m:\Omega \to S^{d-1}≔\left\{x\in R^{d}\;:\;\left|x\right|=1\right\}$ of a rigid ferromagnetic body $\Omega \subset R^{d}$ (d=2, 3) is the minimizer of a (possibly non-convex) minimization problem under a PDE constraint. The following minimization problem, which is the starting point of our previous work  [2] and the present paper, is an example of this problem class:

Problem 1.1 **Reduced Minimization Problem—*RMP***

Let $\Omega \subset R^{d}$, d∈{2,3}, be a bounded Lipschitz domain with boundary Γ and $\varphi^{**}\in C^{1}\left(R^{d},R_{\geq 0}\right)$ be convex. For a given applied field $f\in L^{2}{\left(\Omega \right)}^{d}≔L^{2}\left(\Omega ,R^{d}\right)$ find $m\in A≔\left\{n\in L^{2}{\left(\Omega \right)}^{d}\;:\;\left|n\left(x\right)\right|\leq 1\text{ a.e. in }\Omega \right\}$ that minimizes the energy functional

(1.1) $$E_{f}^{**}\left(m,u\right)≔\int_{\Omega }\varphi^{**}\circ m-\int_{\Omega }f\cdot m+\frac{1}{2}\int_{R^{d}}{\left|\nabla u\right|}^{2}\text{,}$$

where the magnetic potential $u\in {\overset{˙}{BL}}^{1,2}\left(R^{d}\right)$ is related to m through and uniquely defined by

(1.2) $$\mathrm{div}\;\left(\nabla u-m\chi_{\Omega }\right)=0\;\text{in }D{\left(R^{d}\right)}^{\prime }\text{.}$$

Here, $\chi_{\Omega }$ is the characteristic function for the set Ω, and the Beppo-Levi space

(1.3) $${\overset{˙}{BL}}^{1,2}\left(R^{d}\right)=\left\{u\in H_{\ell oc}^{1}\left(R^{d}\right):\;\nabla u\in L^{2}\left(R^{d}\right)\right\}/R$$

is the space of all local $H^{1}$-functions with finite energy, where the constant functions are factored out.

For a discussion of this problem, in particular its relation to more complex models of micromagnetism, we refer to our closely connected earlier work  [2] and to the fundamental paper  [3] on the mathematical analysis of the large-body limit in micromagnetics. On the side of numerical analysis, the present work is intimately linked to  [2] and to [4–7]. We pause to comment on the use of the notation $\varphi^{**}$: In more complex models, the minimization involves a possibly non-convex function φ (in place of $\varphi^{**}$); nevertheless, it is shown in  [3] that replacing φ with its lower convex envelope $\varphi^{**}$ yields a model that still retains relevant macroscopic information.

From a numerical point of view, which is the focus of the present work, Problem 1.1 (RMP) poses several challenges:

(i) The fact that $\varphi^{**}$ is not necessarily *strictly* convex can lead to non-uniqueness of the magnetization m. Even if uniqueness can be ascertained for the continuous problem (this is, for example, the case for so-called “uniaxial materials”, which we will present in Example 1.2) the uniqueness assertion does not necessarily extend to the discrete level. Motivated by techniques of augmented Lagrangian methods, we develop in the present work a consistent stabilization, which allows us to transfer a uniqueness assertion for the continuous problem to the discrete one. In particular, this leads to well-posedness of the discrete problem. A manifestation of the difficulties with uniqueness is that our *a priori* analysis does not control the full $L^{2}$-norm of the error in the magnetization m (cf.  Theorems 4.8 and 4.9).
(ii) The pointwise side constraint |m|≤1 is difficult to realize in practice. Following  [4,6,2] we adopt a penalty approach.
(iii) The energy functional $E_{f}^{**}$ involves a function u that is defined on the full space $R^{d}$ and an integral extending over all of $R^{d}$. A discrete setting requires an appropriate treatment of such functions. In the simplified setting of  [2], the potential u is sought in the space H01(Ω^) for some Ω^⊃Ω with dist(∂Ω^,Ω) sufficiently large. Correspondingly, the integral over $R^{d}$ is replaced with an integral over Ω^. Of course, this procedure introduces an additional modeling error which is neglected in  [4,2] for simplicity. Furthermore, the computational costs are considerably increased owing to the discretization of the large region Ω^∖Ω. In the present work, we circumvent these problems by coupling a finite element method (FEM) to a boundary element method (BEM). The stability and error analysis of this coupling procedure is the principal contribution of this work over  [2].

As mentioned above, the convex function $\varphi^{**}$ may fail to be strictly convex but a uniqueness assertion for the magnetization m may nonetheless be true. We present such a function $\varphi^{**}$ in the following Example 1.2. We will review this uniqueness assertion in the proof of Proposition 3.2, since it sheds light on the requirements for the stabilization in the discrete setting. Our *a priori* error analysis below will in particular cover the case of the function $\varphi^{**}$ of Example 1.2.

Example 1.2 Uniaxial materials, which favor magnetizations m aligned with a so-called “easy axis” $e\in S^{d-1}$ can be modeled with an energy contribution $\int_{\Omega }\varphi \circ m$ in the energy functional $E_{f}$, where the *uniaxial* anisotropy density φ is given by

(1.4) $$\varphi :S^{d-1}⟶R\text{,}\;\varphi \left(x\right)=\frac{1}{2}\left(1-{\left(e\cdot x\right)}^{2}\right)\text{.}$$

As mentioned above, we replace φ in the energy contribution $\int_{\Omega }\varphi \circ m$ with its lower convex envelope $\varphi^{**}$, which then leads to the energy functional of Problem 1.1. In this setting, the lower convex envelope $\varphi^{**}$ is given explicitly as follows for an orthonormal basis $\left\{e,z_{1},\ldots z_{d-1}\right\}$ of $R^{d}$, [4]:

(1.5) $$\varphi^{**}\left(x\right)=\frac{1}{2}\overset{d-1}{\underset{i=1}{\sum }}{\left(x\cdot z_{i}\right)}^{2}\text{,}\;\nabla \varphi^{**}\left(x\right)=\overset{d-1}{\underset{i=1}{\sum }}\left(x\cdot z_{i}\right)z_{i}\text{,}\;\text{for all}\;x\in B^{d}≔\left\{x\in R^{d}:\left|x\right|\leq 1\right\}\text{.}$$

The remainder of the article is organized as follows: In Section  2.1, we recall boundary integral operators and some of their properties in order to reformulate the minimization Problem 1.1 as the minimization Problem 2.4 (also denoted $\left(\overset{˜}{RMP}\right)$) posed on the domain Ω and its boundary Γ≔∂Ω. Since we will work with the saddle point formulations of the continuous and discrete problems, we formulate in Section  3.1 the continuous saddle point problem and show its equivalence with $\left(\overset{˜}{RMP}\right)$. In Section  4.2, we illustrate why a straightforward discretization of the saddle point formulation can lead to instability. Since the overall setting is one of a constrained minimization problem, the key issue is the relation between the kernel of the continuous operator characterizing the constraint and the kernel of its discrete version. The proper relationship can be ensured with suitable consistent stabilization terms, which we present in Section  4.3. Section  4.4 is devoted to a detailed *a priori* error analysis of the stabilized method. We study in detail the case of lowest order discretizations, where we show optimal convergence rates under suitable regularity assumptions. While our stabilization scheme is not restricted to lowest order discretizations, our treatment of the nonlinear terms is particularly well-suited for that setting. We conclude the article in Section  4.5 with numerical examples.

We will use fairly standard notation concerning Sobolev spaces (both integer order spaces $H^{k}\left(\Omega \right)$, $k\in N_{0}$) and fractional Sobolev spaces $H^{1/2}\left(\Gamma \right)$, $H^{-1/2}\left(\Gamma \right)$ as described in [8–11]. We write $H\left(\mathrm{div};R^{d}\right)=\left\{u\in {\left(L^{2}\left(R^{d}\right)\right)}^{d}:\mathrm{div}u\in L^{2}\left(R^{d}\right)\right\}$. We have already introduced the Beppo-Levi space ${\overset{˙}{BL}}^{1,2}\left(R^{d}\right)$ in the statement of Problem 1.1. This space is naturally endowed with the $H^{1}\left(R^{d}\right)$-seminorm. For a comprehensive treatment of this space and the fact that (the natural inclusion of) the test space $D\left(R^{d}\right)$ is dense in ${\overset{˙}{BL}}^{1,2}\left(R^{d}\right)$, we refer the reader to  [12] and  [13, Appendix A].

## 2 The coupled volume–boundary integral equation formulation

### 2.1 Boundary integral operators

In this section we recall some facts from the theory of boundary integral equations and fix notations — we refer the reader to the monographs [8–11] for an extensive discussion of boundary integral operators and boundary element methods.

Let $\Omega \subset R^{d}$ (d=2, 3) be a bounded domain with Lipschitz boundary Γ. We stress that we do not assume that diam(Ω)<1 for the case d=2 as it is often done. We denote the exterior normal vector field on Γ by ν. The interior and exterior trace operators are denoted by $\gamma^{\mathrm{int}}$ and $\gamma^{\mathrm{ext}}$. We define $\partial_{\nu }^{\mathrm{int}}u≔\nu \cdot \gamma^{\mathrm{int}}\nabla u$ and $\partial_{\nu }^{\mathrm{ext}}u≔\nu \cdot \gamma^{\mathrm{ext}}\nabla u$ to be the interior and exterior normal derivative for (sufficiently smooth) functions u on the boundary Γ.

The fundamental solution for Laplace’s equation is

(2.1) $$G\left(x,y\right)=\left\{ \begin{array}{cc} -\frac{1}{2\pi }\log\left|x-y\right| & \text{if }d=2\text{,}\\ \frac{1}{4\pi }\frac{1}{\left|x-y\right|} & \text{if }d=3\text{.} \end{array}\right.$$

For $\phi \in H^{-1/2}\left(\Gamma \right)$ and $u\in H^{1/2}\left(\Gamma \right)$, the *simple layer potential* Vϕ and the *double layer potential* Ku are formally defined by

(2.2) $$\left(V\phi \right)\left(x\right)≔\int_{\Gamma }G\left(x,y\right)\phi \left(y\right)\;dS\left(y\right)\text{,}\;\text{for }x\in R^{d}\setminus \Gamma \text{,}$$

(2.3) $$\left(Ku\right)\left(x\right)≔\int_{\Gamma }\partial_{\nu \left(y\right)}^{\mathrm{int}}G\left(x,y\right)u\left(y\right)\;dS\left(y\right)\text{,}\;\text{for }x\in R^{d}\setminus \Gamma \text{.}$$

The potential operators V and K define solutions of the homogeneous Laplace equation, i.e., for $\phi \in H^{-1/2}\left(\Gamma \right)$ and $u\in H^{1/2}\left(\Gamma \right)$ there holds

(2.4) $$\Delta \left(V\phi \right)\left(x\right)=0\;\text{and}\;\Delta \left(Ku\right)\left(x\right)=0\;\text{for }\;x\in R^{d}\setminus \Gamma \text{.}$$

The *simple layer operator* $V:H^{-1/2}\left(\Gamma \right)\to H^{1/2}\left(\Gamma \right)$, the *double layer operator* $K:H^{1/2}\left(\Gamma \right)\to H^{1/2}\left(\Gamma \right)$, the *adjoint double layer operator* $K^{\prime }:H^{-1/2}\left(\Gamma \right)\to H^{-1/2}\left(\Gamma \right)$, and the *hypersingular operator* $W:H^{1/2}\left(\Gamma \right)\to H^{-1/2}\left(\Gamma \right)$ are formally defined as the compositions of V and K with various trace operators, namely,

(2.5) $$V\phi ≔\gamma^{\mathrm{int}}\left(V\phi \right)=\gamma^{\mathrm{ext}}\left(V\phi \right)\text{,}\;Wu≔-\partial_{\nu }^{\mathrm{int}}\left(Ku\right)=-\partial_{\nu }^{\mathrm{ext}}\left(Ku\right),K^{\prime }\phi ≔\partial_{\nu }^{\mathrm{int}}\left(V\phi \right)-1/2\phi =\partial_{\nu }^{\mathrm{ext}}\left(V\phi \right)+1/2\phi \text{,}\;Ku≔\gamma^{\mathrm{int}}\left(Ku\right)+1/2u=\gamma^{\mathrm{ext}}\left(Ku\right)-1/2u\text{.}$$

For an explicit representation of these operators, we refer to [8]. The operators V and W are furthermore symmetric operators.

By ${\langle u\;;\;\phi \rangle }_{\Gamma }$ we denote the extended $L^{2}\left(\Gamma \right)$-scalar product for functions $\phi \in H^{-1/2}\left(\Gamma \right)$ and $u\in H^{1/2}\left(\Gamma \right)$. We note that $K^{\prime }$ is in fact the adjoint of K with respect to the extended $L^{2}\left(\Gamma \right)$-scalar product. The norms in $H^{-1/2}\left(\Gamma \right)$ and $H^{1/2}\left(\Gamma \right)$ are denoted by ${\left\|\cdot \right\|}_{-1/2,\Gamma }$ and ${\left\|\cdot \right\|}_{1/2,\Gamma }$, respectively. We will work with the function spaces

$$H_{*}^{1/2}\left(\Gamma \right)≔\left\{v\in H^{1/2}\left(\Gamma \right)\;:\;{\langle v\;;\;1\rangle }_{\Gamma }=0\right\},H_{*}^{-1/2}\left(\Gamma \right)≔\left\{\phi \in H^{-1/2}\left(\Gamma \right)\;:\;{\langle \phi \;;\;1\rangle }_{\Gamma }=0\right\}\text{.}$$

In the following two lemmas, we collect some properties of the boundary integral operators that will be needed in the sequel. The following result can be inferred from  [8, Thms. 8.12, 8.21]:

Lemma 2.1 **Ellipticity of V and W**

*Let* $\Omega \subseteq R^{d}$*,* d=2*, 3 be a bounded Lipschitz domain. There exist constants* $c_{1}^{W}$*,* $c_{1}^{V}>0$ *such that*

(2.6) $${\left|u\right|}_{W}^{2}≔{\langle Wu\;;\;u\rangle }_{\Gamma }\geq c_{1}^{W}{\left\|u\right\|}_{1/2,\Gamma }^{2}\;\text{for all}\;u\in H_{*}^{1/2}\left(\Gamma \right)\text{,}$$

(2.7) $${\left\|\phi \right\|}_{V}^{2}≔{\langle V\phi \;;\;\phi \rangle }_{\Gamma }\geq c_{1}^{V}{\left\|\phi \right\|}_{-1/2,\Gamma }^{2}\;\text{for all}\;\phi \in H_{*}^{-1/2}\left(\Gamma \right)\text{.}$$

*For* d=3*the estimate*   (2.7)   *is even valid for all* $\phi \in H^{-1/2}\left(\Gamma \right)$*.*

Lemma 2.2 **Representation Formula and Calderón System**

*Let* $\Omega \subseteq R^{d}$*,* d=2,3 *be a bounded Lipschitz domain. Let the function* $u\in L_{\mathrm{loc}}^{2}\left(\Omega^{\mathrm{ext}}\right)$ *satisfy*

(2.8) $$-\Delta u=0\;\text{in }\Omega^{\mathrm{ext}}≔R^{d}\setminus \bar{\Omega }\text{,}$$

(2.9) $${\left\|\nabla u\right\|}_{L^{2}\left(\Omega^{\mathrm{ext}}\right)}<\infty \text{.}$$

*Then, there exists a constant* $u_{\infty }\in R$ *such that* u *satisfies the following properties*  (i)*–*(vi)*:*

(i) *For every open ball* $B_{R}$ *with* $\bar{\Omega }\subset B_{R}$*there holds* $u\in H^{1}\left(B_{R}\cap \Omega^{\mathrm{ext}}\right)$ *. In particular,* $\gamma^{\mathrm{ext}}u\in H^{1/2}\left(\Gamma \right)$ *is well-defined. Since* −Δu=0 *on* $\Omega^{\mathrm{ext}}$*, also* $\partial_{\nu }^{\mathrm{ext}}u\in H^{-1/2}\left(\Gamma \right)$ *is well-defined. Furthermore, the integration by parts formula holds:*
  $${\langle \nabla u;\nabla \eta \rangle }_{\Omega^{\mathrm{ext}}}=-{\langle \partial_{\nu }^{\mathrm{ext}}u;\eta \rangle }_{\Gamma }\;\forall \eta \in \left\{v\in H^{1}\left(\Omega^{\mathrm{ext}}\right)|\mathrm{supp}\left(v\right)\text{ is compact}\right\}\text{.}$$
(ii) *The radiation condition is satisfied:*
  (2.10) $$u=u_{\infty }+O\left(1/r\right)\text{,}\;r\to \infty \text{.}$$
(iii) *The representation formula is true:*
  (2.11) $$u=K\left(\gamma^{\mathrm{ext}}u\right)-V\left(\partial_{\nu }^{\mathrm{ext}}u\right)+u_{\infty }\;\text{in }\;\Omega^{\mathrm{ext}}\text{.}$$
(iv) *The exterior Calderón system holds:*
  (2.12) $$\gamma^{\mathrm{ext}}u=\left(1/2+K\right)\left(\gamma^{\mathrm{ext}}u\right)-V\left(\partial_{\nu }^{\mathrm{ext}}u\right)+u_{\infty }\text{,}$$
  (2.13) $$\partial_{\nu }^{\mathrm{ext}}u=-W\left(\gamma^{\mathrm{ext}}u\right)+\left(1/2-K^{\prime }\right)\left(\partial_{\nu }^{\mathrm{ext}}u\right)\text{.}$$
(v) ${\langle \partial_{\nu }^{\mathrm{ext}}u;1\rangle }_{\Gamma }=0$*.*
(vi) *Representation of the energy in* $\Omega^{\mathrm{ext}}$*:*
  (2.14) $${\left\|\nabla u\right\|}_{L^{2}\left(\Omega^{\mathrm{ext}}\right)}^{2}=-{\langle \partial_{\nu }^{\mathrm{ext}}u\;;\;u\rangle }_{\Gamma }\text{.}$$

Proof See the Appendix. □

We also need the following auxiliary result:

Lemma 2.3 *Let* $u\in H^{1/2}\left(\Gamma \right)$ *and* $\phi \in H_{*}^{-1/2}\left(\Gamma \right)$ *satisfy*

(2.15) $${\langle \left(1/2-K\right)u+V\phi ;\psi \rangle }_{\Gamma }=0\;\forall \psi \in H_{*}^{-1/2}\left(\Gamma \right)\text{.}$$

*Set* $u_{\infty }≔\left(1/2-K\right)\;u+V\phi \in R$ *. Then, the function* $\overset{˜}{u}≔Ku-V\phi +u_{\infty }$ *satisfies* $\nabla \overset{˜}{u}\in L^{2}\left(\Omega^{\mathrm{ext}}\right)$ *and* $\gamma^{\mathrm{ext}}\overset{˜}{u}=u$ *and* $\partial_{\nu }^{\mathrm{ext}}\overset{˜}{u}=\phi$ *. Furthermore,* $\overset{˜}{u}$ *satisfies*   (2.10)*–*(2.14)   *and, in particular,* $\phi =-Wu+\left(1/2-K^{\prime }\right)\phi$*.*

Proof The condition $\phi \in H_{*}^{-1/2}\left(\Gamma \right)$ implies that the function u^≔Ku−Vϕ satisfies on $\Omega^{\mathrm{ext}}$ the conditions (2.8)–(2.9) and thus has the property (i)–(vi) of Lemma 2.2. Taking the exterior trace on Γ (cf. (2.5)), we obtain with (2.15)

〈γextu^−u;ψ〉Γ=〈(1/2+K)u−Vϕ−u;ψ〉Γ=〈(−1/2+K)u−Vϕ;ψ〉Γ=0∀ψ∈H∗−1/2(Γ).

This implies that (1/2−K)u+Vϕ=u−γextu^≕u∞∈R. The function u˜≔Ku−Vϕ+u∞=u^+u∞ satisfies $\gamma^{\mathrm{ext}}\overset{˜}{u}=u$. Next, $\nabla \overset{˜}{u}\in L^{2}\left(\Omega^{\mathrm{ext}}\right)$ is a consequence of the decay properties of Ku and Vϕ as $\phi \in H_{*}^{-1/2}\left(\Gamma \right)$. Moreover, $\partial_{\nu }^{\mathrm{ext}}\overset{˜}{u}\in H_{*}^{-1/2}\left(\Gamma \right)$ follows from

$${\langle \partial_{\nu }^{\mathrm{ext}}\overset{˜}{u};1\rangle }_{\Gamma }={\langle \partial_{\nu }^{\mathrm{ext}}\left(Ku-V\phi +u_{\infty }\right);1\rangle }_{\Gamma }={\langle -Wu-\left(K^{\prime }-1/2\right)\phi ;1\rangle }_{\Gamma }={\langle \phi ;\left(1/2-K\right)1\rangle }_{\Gamma }=0\text{.}$$

To see $\partial_{\nu }^{\mathrm{ext}}\overset{˜}{u}=\phi$, we first note that Lemma 2.2 gives a second representation of $\overset{˜}{u}$, namely, (2.11):

(2.16) $$\overset{˜}{u}=K\gamma^{\mathrm{ext}}\overset{˜}{u}-V\partial_{\nu }^{\mathrm{ext}}\overset{˜}{u}+{\overset{˜}{u}}_{\infty }=Ku-V\partial_{\nu }^{\mathrm{ext}}\overset{˜}{u}+{\overset{˜}{u}}_{\infty }$$

for some ${\overset{˜}{u}}_{\infty }\in R$. Exploiting the two different representations for $\overset{˜}{u}$, we get $0=-V\left(\partial_{\nu }^{\mathrm{ext}}\overset{˜}{u}-\phi \right)+{\overset{˜}{u}}_{\infty }-u_{\infty }$ on $\Omega^{\mathrm{ext}}$; applying $\partial_{\nu }^{\mathrm{ext}}$ yields (cf. (2.5)) $0=\left(1/2-K^{\prime }\right)\left(\partial_{\nu }^{\mathrm{ext}}\overset{˜}{u}-\phi \right)$. The assertion $\partial_{\nu }^{\mathrm{ext}}\overset{˜}{u}-\phi =0$ is obtained from $\partial_{\nu }^{\mathrm{ext}}\overset{˜}{u}-\phi \in H_{*}^{-1/2}\left(\Gamma \right)$ and the fact that $1/2-K^{\prime }$ is one-to-one on $H_{*}^{-1/2}\left(\Gamma \right)$ (cf., e.g.,  [14, Thm. 4.2] for the case d=2 and  [14, Thm. 3.3] for d≥3). □

### 2.2 Reformulation of (RMP) using boundary integrals

With the boundary integral operators in hand, we can rephrase the minimization Problem 1.1, which involves the function u as a function on the full space $R^{d}$, as a problem posed on the bounded domain Ω and the boundary Γ=∂Ω. This is achieved with the energy representation formula (2.14). In Proposition 2.5, we will formally show the equivalence of Problems (RMP) and $\left(\overset{˜}{RMP}\right)$.

Problem 2.4 **$\overset{˜}{RMP}$**

Find a function

$$u\in H_{*}^{1}\left(\Omega \right)≔\left\{v\in H^{1}\left(\Omega \right)\;:\;{\langle v\;;\;1\rangle }_{\Gamma }=0\right\}\text{,}$$

a magnetization state m∈A, and a function $\phi \in H_{*}^{-1/2}\left(\Gamma \right)$ that minimize the energy functional

(2.17) $${\overset{˜}{E}}_{f}^{**}\left(u,m,\phi \right)≔\int_{\Omega }\varphi^{**}\circ m-\int_{\Omega }f\cdot m+\frac{1}{2}\int_{\Omega }{\left|\nabla u\right|}^{2}-\frac{1}{2}{\langle \phi \;;\;u\rangle }_{\Gamma }\text{,}$$

under the side constraints

(2.18) $${\langle \nabla u-m\;;\;\nabla \eta \rangle }_{\Omega }-{\langle \phi \;;\;\eta \rangle }_{\Gamma }=0\;\text{for all}\;\eta \in D\left(R^{d}\right)\text{,}$$

(2.19) $${\langle V\phi +\left(1/2-K\right)\left(\gamma^{\mathrm{int}}u\right)\;;\;\psi \rangle }_{\Gamma }=0\;\text{for all}\;\psi \in H_{*}^{-1/2}\left(\Gamma \right)\text{,}$$

where ${\langle \cdot \;;\;\cdot \rangle }_{\Omega }$ denotes the $L^{2}\left(\Omega \right)$  scalar-product.

Proposition 2.5 Problem  1.1(RMP) *and*   Problem  2.4$\left(\overset{˜}{RMP}\right)$ *are equivalent in the following sense:*

(i) *Let* $\left(u,m\right)\in {\overset{˙}{BL}}^{1,2}\left(R^{d}\right)\times A$ *be a solution of* (RMP) *. Let* $u_{\Gamma }≔{\langle u;1\rangle }_{\Gamma }/\left|\Gamma \right|$ *be the integral mean of* u *over* Γ *. Then* $\left(u{|}_{\Omega }-u_{\Gamma },m,\partial_{\nu }^{\mathrm{ext}}u\right)$ *solves* $\left(\overset{˜}{RMP}\right)$*.*
(ii) *Let* $\left(u,m,\phi \right)\in H_{*}^{1}\left(\Omega \right)\times A\times H_{*}^{-1/2}\left(\Gamma \right)$ *be a solution of* $\left(\overset{˜}{RMP}\right)$ *. Then,* $u_{\infty }≔\left(1/2-K\right)u+V\phi \in R$ *and* $\left(\overset{˜}{u},m\right)$ *solves* (RMP)*, where* $\overset{˜}{u}$ *is defined by*
  (2.20) $$\overset{˜}{u}\left(x\right)≔\left\{ \begin{array}{cc} u\left(x\right) & \text{if }x\in \bar{\Omega }\text{,}\\ \left(K\gamma^{\mathrm{int}}u\right)\left(x\right)-\left(V\phi \right)\left(x\right)+u_{\infty } & \text{if }x\in R^{d}\setminus \bar{\Omega }\text{.} \end{array}\right.$$

*Moreover, the relaxed minimization problem* $\left(\overset{˜}{RMP}\right)$ *has solutions.*

Proof **Step 1:** Suppose that $\left(u,m\right)\in {\overset{˙}{BL}}^{1,2}\left(R^{d}\right)\times A$ satisfies the side constraint (1.2) of (RMP). We show in this step that $\left(u{|}_{\Omega }-u_{\Gamma },m,\partial_{\nu }^{\mathrm{ext}}u\right)\in H_{*}^{1}\left(\Omega \right)\times A\times H_{*}^{-1/2}\left(\Gamma \right)$ satisfies the side constraints (2.18)–(2.19) of $\left(\overset{˜}{RMP}\right)$ and that $E_{f}^{**}\left(u,m\right)={\overset{˜}{E}}_{f}^{**}\left(u{|}_{\Omega }-u_{\Gamma },m,\partial_{\nu }^{\mathrm{ext}}u\right)$.

Since ${\overset{˙}{BL}}^{1,2}\left(R^{d}\right)$ is a factor space in which the constant functions are factored out, we may choose a representative with ${\langle u,1\rangle }_{\Gamma }=0$, i.e.,  $u{|}_{\Omega }\in H_{*}^{1}\left(\Omega \right)$. Eq. (1.2) implies Δu=0 in $\Omega^{\mathrm{ext}}$. Since $\nabla u\in L^{2}\left(\Omega^{\mathrm{ext}}\right)$, we obtain from Lemma 2.2 (i), (v), and (vi) that

(2.21) $${\langle \partial_{\nu }^{\mathrm{ext}}u;\eta \rangle }_{\Gamma }=-{\langle \nabla u;\nabla \eta \rangle }_{\Omega^{\mathrm{ext}}}\;\forall \eta \in D\left(R^{d}\right)\text{,}$$

(2.22) $$\partial_{\nu }^{\mathrm{ext}}u\in H_{*}^{-1/2}\left(\Gamma \right)\text{,}$$

(2.23) $$-{\langle \partial_{\nu }^{\mathrm{ext}}u;u\rangle }_{\Gamma }={\left\|\nabla u\right\|}_{L^{2}\left(\Omega^{\mathrm{ext}}\right)}^{2}\text{.}$$

Next, the following calculation shows that $\left(u{|}_{\Omega }-u_{\Gamma },m,\partial_{\nu }^{\mathrm{ext}}u\right)$ satisfies (2.18):

(2.24) $$0={\langle \nabla u-m\chi_{\Omega }\;;\;\nabla \eta \rangle }_{R^{d}}={\langle \nabla u-m\;;\;\nabla \eta \rangle }_{\Omega }-{\langle \partial_{\nu }^{\mathrm{ext}}u\;;\;\eta \rangle }_{\Gamma }\;\text{for all}\;\eta \in D\left(R^{d}\right)\text{.}$$

Since Lemma 2.2 is applicable for $u{|}_{\Omega^{\mathrm{ext}}}$, the representation (2.12) holds. As $u\in {\overset{˙}{BL}}^{1,2}\left(R^{d}\right)$ is continuous across Γ, we have $\gamma^{\mathrm{int}}u=\gamma^{\mathrm{ext}}u$ and therefore (2.19) holds. Finally, (2.23) implies

$$\int_{\Omega }{\left|\nabla u\right|}^{2}-{\langle \partial_{\nu }^{\mathrm{ext}}u\;;\;u\rangle }_{\Gamma }=\int_{R^{d}}{\left|\nabla u\right|}^{2}\text{,}$$

and hence $E_{f}^{**}\left(u,m\right)={\overset{˜}{E}}_{f}^{**}\left(u{|}_{\Omega }-u_{\Gamma },m,\partial_{\nu }^{\mathrm{ext}}u\right)$.

**Step 2:** Suppose that $\left(u,m,\phi \right)\in H_{*}^{1}\left(\Omega \right)\times A\times H_{*}^{-1/2}\left(\Gamma \right)$ satisfies the side constraints (2.18)–(2.19) of $\left(\overset{˜}{RMP}\right)$. With $\overset{˜}{u}$ from  (2.20), we show in this step that $\left(\overset{˜}{u},m\right)\in {\overset{˙}{BL}}^{1,2}\left(R^{d}\right)\times A$ satisfies the side constraint (1.2) of (RMP) and that ${\overset{˜}{E}}_{f}^{**}\left(u,m,\phi \right)=E_{f}^{**}\left(\overset{˜}{u},m\right)$.

By Lemma 2.3, we can find $u_{\infty }\in R$ such that the function $\overset{˜}{u}$ defined in (2.20) is continuous across Γ, i.e.,  $\gamma^{\mathrm{ext}}\overset{˜}{u}=\gamma^{\mathrm{int}}u$. Furthermore, it holds $\overset{˜}{u}\in {\overset{˙}{BL}}^{1,2}\left(R^{d}\right)$ and $\partial_{\nu }^{\mathrm{ext}}\overset{˜}{u}=\phi$. Using this identity and $u=\overset{˜}{u}$ in Ω in (2.18) gives

(2.25) $${\langle \nabla \overset{˜}{u}-m\chi_{\Omega }\;;\;\nabla \eta \rangle }_{R^{d}}={\langle \nabla u-m\;;\;\nabla \eta \rangle }_{\Omega }-{\langle \partial_{\nu }^{\mathrm{ext}}\overset{˜}{u}\;;\;\eta \rangle }_{\Gamma }=0\;\text{for all}\;\eta \in D\left(R^{d}\right)\text{.}$$

Moreover, Lemma 2.2 gives

$$\int_{\Omega }{\left|\nabla u\right|}^{2}-{\langle \phi \;;\;u\rangle }_{\Gamma }=\int_{\Omega }{\left|\nabla \overset{˜}{u}\right|}^{2}-{\langle \partial_{\nu }^{\mathrm{ext}}\overset{˜}{u}\;;\;\overset{˜}{u}\rangle }_{\Gamma }=\int_{R^{d}}{\left|\nabla \overset{˜}{u}\right|}^{2}\text{,}$$

and hence ${\overset{˜}{E}}_{f}^{**}\left(u,m,\phi \right)=E_{f}^{**}\left(\overset{˜}{u},m\right)$.

**Step 3:** Let $\left(u,m\right)\in {\overset{˙}{BL}}^{1,2}\left(R^{d}\right)\times A$ be a minimizer of (RMP) and $\left(u^{\prime },m^{\prime },\phi^{\prime }\right)\in H_{*}^{1}\left(\Omega \right)\times A\times H_{*}^{-1/2}\left(\Gamma \right)$ be a minimizer of $\left(\overset{˜}{RMP}\right)$. From Steps 1 and 2, it follows that $E_{f}^{**}\left(u,m\right)={\overset{˜}{E}}_{f}^{**}\left(u^{\prime },m^{\prime },\phi^{\prime }\right)$. This shows the equivalence of (RMP) and $\left(\overset{˜}{RMP}\right)$.

**Step 4:**[3] proves that (RMP) has solutions. Since (RMP) and $\left(\overset{˜}{RMP}\right)$ are equivalent, this proves that $\left(\overset{˜}{RMP}\right)$ has solutions as well. □

Various FEM–BEM coupling methods could be formulated starting from $\left(\overset{˜}{RMP}\right)$ following the techniques proposed and discussed in  [15–19]. Here, we focus on the symmetric FEM–BEM coupling due to  [18]. In the symmetric FEM–BEM coupling, the second equation of the exterior Calderón system (2.13),

(2.26) $$\phi =-W\left(\gamma^{\mathrm{int}}u\right)+\left(1/2-K^{\prime }\right)\phi \text{,}$$

is substituted for the variable ϕ in (2.17) and (2.18).

## 3 The continuous problem

### 3.1 The saddle point problem

En route to a numerical scheme, we reformulate in this section the minimization problem $\left(\overset{˜}{RMP}\right)$ as a saddle point problem, denoted *(SPP)*. In the following Proposition 3.2, we show their equivalence and the unique solvability in the case of uniaxial materials of Example 1.2. One of our reasons for presenting the uniqueness assertions of Problem 3.1 on the continuous level is to be able to highlight the need of a suitable stabilization for the discrete setting in Theorem 4.6.

Problem 3.1 **SPP**

Find $u=\left(u,m,\phi \right)\in X≔H_{*}^{1}\left(\Omega \right)\times L^{2}{\left(\Omega \right)}^{d}\times H_{*}^{-1/2}\left(\Gamma \right)$, $p=\left(p,\zeta \right)\in M≔H_{*}^{1}\left(\Omega \right)\times H_{*}^{-1/2}\left(\Gamma \right)$ and $\lambda_{m}\in L^{2}\left(\Omega ,R_{\geq 0}\right)$ such that

(3.1) $$a\left(u;v\right)+b\left(v;p\right)={\langle f\;;\;n\rangle }_{\Omega }\;\text{for all}\;v=\left(v,n,\psi \right)\in X\text{,}$$

(3.2) b(u;q)=0for allq=(q,θ)∈M,

(3.3) $$\lambda_{m}\left(1-\left|m\right|\right)=0$$

under the constraint |m(x)|≤1 almost everywhere in Ω; here

(3.4) $$a\left(u;v\right)≔{\langle \nabla u\;;\;\nabla v\rangle }_{\Omega }+{\langle Wu+1/2\left(K^{\prime }-1/2\right)\phi \;;\;v\rangle }_{\Gamma }+{\langle \nabla \varphi^{**}\circ m+\lambda_{m}m\;;\;n\rangle }_{\Omega }+1/2{\langle \left(K-1/2\right)u\;;\;\psi \rangle }_{\Gamma }\text{,}$$

(3.5) $$b\left(u;q\right)≔-{\langle \nabla u-m\;;\;\nabla q\rangle }_{\Omega }-{\langle Wu+\left(K^{\prime }-1/2\right)\phi \;;\;q\rangle }_{\Gamma }+{\langle V\phi -\left(K-1/2\right)u\;;\;\theta \rangle }_{\Gamma }\text{.}$$

Proposition 3.2 **Equivalence of (SPP) and $\left(\overset{˜}{RMP}\right)$ & (unique) Solvability**

*The following statements*  (i)*–*(iii)  *are true:*

(i) *The minimization problem* $\left(\overset{˜}{RMP}\right)$ *and the saddle point problem* (SPP) *are equivalent in the following sense: for every solution* (u,m,ϕ) *of* $\left(\overset{˜}{RMP}\right)$*there exist* p*,* $\lambda_{m}$ *such that* $\left(u,m,\phi ,p,\lambda_{m}\right)$ *solves* (SPP) *and conversely, the components* (u,m,ϕ) *of a solution* $\left(u,m,\phi ,p,\lambda_{m}\right)$ *of* (SPP) *solve* $\left(\overset{˜}{RMP}\right)$*.*
(ii) *The magnetic potential* u*, its exterior normal derivative* ϕ*, and the Lagrange multipliers* p *and* ζ *are uniquely determined in* (SPP)*.*
(iii) *If* $\varphi^{**}$ *is given as in*Example  1.2   *(“uniaxial case”), then problems* $\left(\overset{˜}{RMP}\right)$ *and* (SPP) *are uniquely solvable.*

Proof Proof of (i): [3] shows the equivalence of the minimization problem *(RMP)* with the corresponding Euler–Lagrange equation (3.6a) and the side constraints (3.6b) and (3.6c): Find $\left(u,m\right)\in {\overset{˙}{BL}}^{1,2}\left(R^{d}\right)\times L^{2}{\left(\Omega \right)}^{d}$ and $\lambda_{m}\in L^{2}\left(\Omega ,R_{\geq 0}\right)$ such that

(3.6a) $${\langle \nabla u+\nabla \varphi^{**}\circ m+\lambda_{m}m\;;\;n\rangle }_{\Omega }={\langle f\;;\;n\rangle }_{\Omega }\;\text{for all}\;n\in L^{2}{\left(\Omega \right)}^{d}\text{,}$$

(3.6b) $${\langle \nabla u-m\chi_{\Omega }\;;\;\nabla \eta \rangle }_{R^{d}}=0\;\text{for all}\;\eta \in D\left(R^{d}\right)\text{,}$$

(3.6c) $$\lambda_{m}\left(x\right)\left(1-\left|m\left(x\right)\right|\right)=0\;\text{for almost every}\;x\in \Omega \text{.}$$

We show the equivalence of (SPP) with (3.6). To that end let $\left(u,m,\lambda_{m}\right)$ be a solution of (3.6). Recalling Eq. (2.26) the equivalence of (3.6b) and (3.2)  can be shown similarly as in the proof of Proposition 2.5. Setting $p=u{|}_{\Omega }$ and $\zeta =\frac{1}{2}\phi$ and, of course, $\phi =\partial_{\nu }^{\mathrm{ext}}u$ shows that the tuple $\left(u{|}_{\Omega },m,\phi ,\lambda_{m};p,\zeta \right)$ satisfies Eq. (3.1).

Consider now in turn a solution $\left(u,m,\phi ,\lambda_{m};p,\zeta \right)$ of (SPP). We first show p=u and $\zeta =\frac{1}{2}\phi$. Subtract Eq. (3.2) tested with q=(0,ψ) and multiplied with 1/2 from Eq. (3.1) tested with v=(0,0,ψ) and set ψ=ϕ−2ζ afterward. This gives

(3.7) $$\frac{1}{2}{\langle \left(K-1/2\right)\left(u-p\right)\;;\;\phi -2\zeta \rangle }_{\Gamma }-\frac{1}{4}{\langle V\left(\phi -2\zeta \right)\;;\;\phi -2\zeta \rangle }_{\Gamma }=0\text{.}$$

Subtracting this equation from Eq. (3.1) tested with v=(u−p,0,0) leads us to

(3.8) $${\left\|\nabla \left(u-p\right)\right\|}_{\Omega }^{2}+{\left|u-p\right|}_{W}^{2}+\frac{1}{4}{\left\|\phi -2\zeta \right\|}_{V}^{2}=0\text{,}$$

from which we deduce the claimed p=u and $\zeta =\frac{1}{2}\phi$. Here, ${\left\|\cdot \right\|}_{\Omega }$ denotes the usual norm in $L^{2}\left(\Omega \right)$. With p=u Eq. (3.1) tested with v=(0,n,0) results in Eq. (3.6a).

Proof of (ii): To prove uniqueness of the magnetic potential u and its exterior normal derivative ϕ, we follow the lines of [4]. Let $u_{i}=\left(u_{i},m_{i},\phi_{i}\right)\in X$, $p_{i}=\left(p_{i},\zeta_{i}\right)\in M$ and $\lambda_{m_{i}}\in L^{2}\left(\Omega ;R_{\geq 0}\right)$, i=1,2, be two solutions of (SPP). Subtracting Eqs. (3.1) and (3.2) yields together with the test functions $v=\left(u_{2}-u_{1},m_{2}-m_{1},\phi_{2}-\phi_{1}\right)\in X$ and $q=\left(p_{2}-p_{1},\zeta_{2}-\zeta_{1}\right)\in M$

(3.9) $${\left\|\nabla \left(u_{2}-u_{1}\right)\right\|}_{\Omega }^{2}+{\left|u_{2}-u_{1}\right|}_{W}^{2}+{\langle \nabla \varphi^{**}\circ m_{2}-\nabla \varphi^{**}\circ m_{1}\;;\;m_{2}-m_{1}\rangle }_{\Omega }+{\langle \lambda_{m_{2}}m_{2}-\lambda_{m_{1}}m_{1}\;;\;m_{2}-m_{1}\rangle }_{\Omega }+{\langle \left(K-1/2\right)\left(u_{2}-u_{1}\right)\;;\;\phi_{2}-\phi_{1}\rangle }_{\Gamma }=0\text{,}$$

where the last term can be replaced by ${\left\|\phi_{2}-\phi_{1}\right\|}_{V}^{2}$ in view of (3.2). From the convexity of $\varphi^{**}$, we get the non-negativity of the third term, and pointwise non-negativity of the fourth term was proved in [4]. Hence, all terms vanish, and we deduce $u_{2}=u_{1}$ and $\phi_{2}=\phi_{1}$.

To show the uniqueness of p and ζ, let two solutions $\left(u,m,\phi ,\lambda_{m};p_{i},\zeta_{i}\right)\in X\times M$, i=1,2, be given and set u=(u,m,ϕ) and $p_{i}=\left(p_{i},\zeta_{i}\right)$, i=1,2. From (3.1), we get

(3.10) $$b\left(v,p_{2}-p_{1}\right)=0\;\text{for all}\;v=\left(v,n,\psi \right)\in X\text{,}$$

and the desired conclusion $p_{1}=p_{2}$ follows from the fact that the bilinear form b satisfies an inf–sup condition. Indeed, with the norms

(3.11) $${\left\|u\right\|}_{X}^{2}≔{\left\|\nabla u\right\|}_{\Omega }^{2}+{\left\|m\right\|}_{\Omega }^{2}+{\left\|\phi \right\|}_{-1/2,\Gamma }^{2}\;\text{and}\;{\left\|p\right\|}_{M}^{2}≔{\left\|\nabla p\right\|}_{\Omega }^{2}+{\left\|\zeta \right\|}_{-1/2,\Gamma }^{2}\text{,}$$

we get for arbitrary p=(p,ζ)∈M∖{0} by Lemma 2.1

(3.12) $$\underset{u\in X\setminus \left\{0\right\}}{\sup}\frac{\left|b\left(u;p\right)\right|}{{\left\|u\right\|}_{X}{\left\|p\right\|}_{M}}\geq \frac{\left|b\left(-p,0,\zeta ;p,\zeta \right)\right|}{{\left\|\left(-p,0,\zeta \right)\right\|}_{X}{\left\|\left(p,\zeta \right)\right\|}_{M}}=\frac{1}{\overset{2}{\underset{M}{\left\|\left(p,\zeta \right)\right\|}}}\left\{\overset{2}{\underset{\Omega }{\left\|\nabla p\right\|}}+{\langle Wp\;;\;p\rangle }_{\Gamma }+{\langle V\zeta \;;\;\zeta \rangle }_{\Gamma }\right\}\geq \min\left\{1,\overset{V}{\underset{1}{c}}\right\}>0\text{.}$$

This implies

(3.13) $$\underset{p\in M\setminus \left\{0\right\}}{\inf}\underset{u\in X\setminus \left\{0\right\}}{\sup}\frac{\left|b\left(u;p\right)\right|}{{\left\|u\right\|}_{X}{\left\|p\right\|}_{M}}\geq \min\left\{1,\overset{V}{\underset{1}{c}}\right\}>0\text{.}$$

Proof of (iii): This assertion was proved in  [6]. We repeat here the essential arguments to give an idea of what the key properties are that the stabilization term for the discrete method should have. As explained above, Eq. (3.9) yields ${\langle \nabla \varphi^{**}\circ m_{2}-\nabla \varphi^{**}\circ m_{1}\;;\;m_{2}-m_{1}\rangle }_{\Omega }=0$. Using the explicit formula for $\nabla \varphi^{**}$ given in Example 1.2, we get

(3.14) $$\overset{d-1}{\underset{i=1}{\sum }}{\left\|\left(m_{2}-m_{1}\right)\cdot z_{i}\right\|}_{\Omega }^{2}=0\text{.}$$

Eq. (3.2) together with the knowledge of uniquely determined u and ϕ (see (3.9)), gives by linearity $b\left(0,m_{2}-m_{1},0;q\right)=0$ for all q∈M. In other words, there holds $\left(0,m_{2}-m_{1},0\right)\in \ker\;b\subseteq X$. From this, we deduce

(3.15) $$\mathrm{div}\;\left(m_{2}-m_{1}\right)\chi_{\Omega }=0\;\text{in}\;D{\left(R^{d}\right)}^{\prime }\text{.}$$

Combining (3.14) and (3.15) implies $m_{2}-m_{1}=0$: For sufficiently smooth magnetizations, this follows by classical calculus. In the present setting of distributions, smoothing arguments have to be employed as shown in [20, Satz 2.12] or [21, Lemma 14]. This concludes the proof. □

### 3.2 Penalization

The pointwise side constraint |m(x)|≤1 is difficult to enforce numerically. We will therefore relax this condition using a penalty method as originally used in  [4] and later also in [6,7]. We assume from now on that $\varphi^{**}$ is the restriction to $B^{d}$ of a convex and continuous differentiable function defined on the full space $R^{d}$.

Given a function $\varepsilon \in L^{\infty }\left(\Omega ,R_{>0}\right)$ the penalized problem $\left(RMP_{\varepsilon }\right)$ is:

Problem 3.3 **Penalized Problem $\left(RMP_{\varepsilon }\right)$**

Find a minimizer $u\in H_{*}^{1}\left(\Omega \right)$, $m\in L^{2}{\left(\Omega \right)}^{d}$ and $\phi \in H_{*}^{-1/2}\left(\Gamma \right)$ of

(3.16) $$E_{f,\varepsilon }^{**}\left(u,m,\phi \right)={\overset{˜}{E}}_{f}^{**}\left(u,m,\phi \right)+\frac{1}{2}\int_{\Omega }\frac{{\left(\left|m\right|-1\right)}_{+}^{2}}{\varepsilon }\text{,}$$

under the side constraints (2.18) and (2.19).

Later on, the penalization parameter ε will be related to the local mesh size in the discrete version of (3.16). We mention that $E_{f,\varepsilon }^{**}$ is convex, continuous, Gâteaux differentiable, and coercive. In particular, the direct method of the calculus of variations proves that $\left(RMP_{\varepsilon }\right)$ has solutions, and Proposition 3.2 holds accordingly. Related arguments can be found in [22,2,6,4,7]. We omit the details.

## 4 The discrete problem

### 4.1 Notation

Let $T≔\left\{K_{1},\ldots ,K_{M}\right\}$ denote an affine, regular, γ-shape regular triangulation of Ω and let $T{|}_{\Gamma }$ be the set of all edges (d=2) or faces (d=3) of elements of T on Γ. The spaces of scalar-valued or vector-valued polynomials of (total) degree k on an element K are denoted $P^{k}\left(K\right)$ and $P^{k}{\left(K\right)}^{d}$. We introduce the linear space

(4.1) $$S_{*}^{1,1}\left(T\right)=\left\{u\in H_{*}^{1}\left(\Omega \right)\;:\;\forall K\in T:u{|}_{K}\in P^{1}\left(K\right)\right\}$$

of all T-piecewise affine, globally continuous scalar fields with vanishing integral mean on Γ. By

(4.2) $$S^{0,0}\left(T\right)=\left\{v\in L^{2}\left(\Omega \right)\;:\;\forall K\in T:v{|}_{K}\in P^{0}\left(K\right)\right\}\;\text{and}$$

(4.3) $$S^{0,0}{\left(T\right)}^{d}=\left\{m\in L^{2}{\left(\Omega \right)}^{d}\;:\;\forall K\in T:m{|}_{K}\in P^{0}{\left(K\right)}^{d}\right\}$$

we denote the linear space of all T-piecewise constant scalar fields and vector fields, respectively. The linear space of all $T{|}_{\Gamma }$-piecewise constant scalar fields with vanishing integral mean is denoted by

(4.4) $$S_{*}^{0,0}\left(T{|}_{\Gamma }\right)≔\left\{\phi \in H_{*}^{-1/2}\left(\Gamma \right)\;:\;\forall e\in T{{|}_{\Gamma }:\phi |}_{e}\in P^{0}\left(e\right)\right\}\text{.}$$

In addition we use the abbreviations $X_{N}≔S_{*}^{1,1}\left(T\right)\times S^{0,0}{\left(T\right)}^{d}\times S_{*}^{0,0}\left(T{|}_{\Gamma }\right)\subseteq X$ and $M_{N}≔S_{*}^{1,1}\left(T\right)\times S_{*}^{0,0}\left(T{|}_{\Gamma }\right)\subseteq M$.

### 4.2 An unstable saddle point formulation

We formulate now a discrete version of the saddle point problem (SPP). The starting point is the minimization of the penalized energy functional $E_{f,\varepsilon }^{**}\left(u\right)$ on the discrete space $X_{N}$. To be precise, the minimization problem $\left(RMP_{\varepsilon }^{N}\right)$ is: Find $u_{N}=\left(u_{N},m_{N},\phi_{N}\right)\in X_{N}$ such that $E_{f,\varepsilon }^{**}$ is minimized under the side constraint

(4.5) $$b\left(u_{N};q_{N}\right)=0\;\text{for all}\;q_{N}=\left(q_{N},\theta_{N}\right)\in M_{N}\text{.}$$

The Lagrangian $L_{\varepsilon }$ associated with this constrained minimization problem is, with $p_{N}=\left(p_{N},\zeta_{N}\right)\in M_{N}$,

(4.6) $$L_{\varepsilon }\left(u_{N};p_{N}\right)=E_{f,\varepsilon }^{**}\left(u_{N}\right)+b\left(u_{N};p_{N}\right)\text{,}\;\left(u_{N};p_{N}\right)\in X_{N}\times M_{N}\text{.}$$

The solution of the constrained minimization problem is the stationary point of the Lagrangian $L_{\varepsilon }$. If we choose the penalization parameter ε to be a T-piecewise constant function, we can compute the derivatives of $L_{\varepsilon }$ explicitly. This leads us to the following formulation.

Problem 4.1 **$SPP_{\varepsilon }^{N}$**

Let $\varepsilon \in S^{0,0}\left(T\right)$ and ε>0. Find $\left(u_{N};p_{N}\right)=\left(u_{N},m_{N},\phi_{N};p_{N},\zeta_{N}\right)\in X_{N}\times M_{N}$ such that

(4.7) $$a_{N}\left(u_{N};v\right)+b\left(v;p_{N}\right)={\langle f\;;\;n\rangle }_{\Omega }\;\text{for all}\;v=\left(v,n,\psi \right)\in X_{N}\text{,}$$

(4.8) $$b\left(u_{N};q\right)=0\;\text{for all}\;q=\left(q,\theta \right)\in M_{N}\text{,}$$

where we set

(4.9) $$a_{N}\left(u_{N};v\right)≔{\langle \nabla u_{N}\;;\;\nabla v\rangle }_{\Omega }+{\langle Wu_{N}+1/2\left(K^{\prime }-1/2\right)\phi_{N}\;;\;v\rangle }_{\Gamma }+{\langle \nabla \varphi^{**}\circ m_{N}+\lambda_{N}m_{N}\;;\;n\rangle }_{\Omega }+\frac{1}{2}{\langle \left(K-1/2\right)u_{N}\;;\;\psi \rangle }_{\Gamma }\text{,}$$

(4.10) $$\lambda_{N}≔\frac{{\left(\left|m_{N}\right|-1\right)}_{+}}{\varepsilon \left|m_{N}\right|}\text{.}$$

Compared with the continuous formulation in Problem 3.1, the main difference is that the continuous Lagrange multiplier $\lambda_{m}\in L^{2}\left(\Omega ,R_{\geq 0}\right)$, characterized by the condition (3.3), is replaced by the term (4.10).

Since the minimization problem $\left(RMP_{\varepsilon }^{N}\right)$ has solutions, it is easy to show via the Euler–Lagrange equation that $\left(SPP_{\varepsilon }^{N}\right)$ has solutions as well. Here, the existence and uniqueness of the Lagrange parameters $p_{N}$ and $\zeta_{N}$ follow from a discrete inf–sup condition of the bilinear form b in the same way as in the proof of Proposition 3.2. Reviewing the arguments of this proof also shows the existence and uniqueness of $u_{N}$ and $\phi_{N}$. However, uniqueness of the magnetization $m_{N}$ cannot be ensured in the same way as in the proof of Proposition 3.2, since $\ker_{N}\;b⊈\ker\;b$, where

(4.11) kerb≔{u∈X:b(u;q)=0for allq∈M}⊆Xand

(4.12) $$\ker_{N}\;b≔\left\{u_{N}\in X_{N}\;:\;b\left(u_{N};q\right)=0\;\text{for all}\;q\in M_{N}\right\}\subseteq X_{N}\text{.}$$

This lack of uniqueness expresses the fact that the discrete formulation is unstable, cf.  [4,7]. In the next section, we show how to enforce stability in the discrete case by adding a suitable stabilization term. We close this section by making more explicit some properties of kerb:

Lemma 4.2 *A triple* u=(u,m,ϕ)∈kerb *satisfies:*

(i) ∇u−m∈H(div;Ω) *and additionally* $\mathrm{div}\left(\nabla u-m\right)=0\in L^{2}\left(\Omega \right)$ *;*
(ii) $\left(\nabla u-m\right)\cdot \nu =\phi \in H_{*}^{-1/2}\left(\Gamma \right)$*, where* ν *denotes the exterior normal vector on* Γ*.*

Proof u∈kerb implies $\mathrm{div}\left(\nabla u-m\right)=0\in H^{-1}\left(\Omega \right)$ so that $\mathrm{div}\left(\nabla u-m\right)=0\in L^{2}\left(\Omega \right)$ follows, which gives us ∇u−m∈H(div;Ω). Hence, $\left(\nabla u-m\right)\cdot \nu \in H^{-1/2}\left(\Gamma \right)$. To see $\left(\nabla u-m\right)\cdot \nu \in H_{*}^{-1/2}\left(\Gamma \right)$, we note ${\langle \left(\nabla u-m\right)\cdot \nu ;1\rangle }_{\Gamma }=-{\langle \mathrm{div}\left(\nabla u-m\right);1\rangle }_{\Omega }=0$. Finally, the assertion (∇u−m)⋅ν−ϕ=0 is seen as follows: First, the condition ${\langle V\phi -\left(K-1/2\right)u;\theta \rangle }_{\Gamma }=0$ for all $\theta \in H_{*}^{-1/2}\left(\Gamma \right)$ implies by Lemma 2.3 the relation $\phi =-Wu+\left(1/2-K^{\prime }\right)\phi$. Thus, in view of div(∇u−m)=0, we obtain

$$0=-{\langle \left(\nabla u-m\right)\cdot \nu ;q\rangle }_{\Gamma }-{\langle Wu+\left(K^{\prime }-1/2\right)\phi ;q\rangle }_{\Gamma }=-{\langle \left(\nabla u-m\right)\cdot \nu -\phi ;q\rangle }_{\Gamma }\;\forall q\in H_{*}^{1/2}\left(\Gamma \right)\text{.}$$

Since $\left(\nabla u-m\right)\cdot \nu -\phi \in H_{*}^{-1/2}\left(\Gamma \right)$, this implies (∇u−m)⋅ν−ϕ=0. □

### 4.3 A stable saddle point formulation

In this section, we present a consistent stabilized formulation. The stabilization may ensure uniqueness of the magnetization $m_{N}$ in a solution $\left(u_{N},m_{N},\phi_{N};p_{N},\zeta_{N}\right)$; in other words, the formulation provides unique solvability of the modified saddle point formulation.

We introduce the augmented Lagrangian as

(4.13) $$L_{\varepsilon }^{aug}\left(u_{N};p_{N}\right)≔E_{f,\varepsilon }^{**}\left(u_{N}\right)+b\left(u_{N};p_{N}\right)+\frac{1}{2}\sigma \left(u_{N};u_{N}\right)\text{,}$$

where the stabilizing bilinear form $\sigma :\left(\ker b+X_{N}\right)\times \left(\ker b+X_{N}\right)\to R$ is defined by

(4.14) $$\sigma \left(u;v\right)≔\underset{e\in E_{\Omega }\left(T\right)}{\sum }h_{e}{\langle {\left[\left(\nabla u-m\right)\cdot \nu \right]}_{e}\;;\;{\left[\left(\nabla v-n\right)\cdot \nu \right]}_{e}\rangle }_{e}+\underset{e\in T{|}_{\Gamma }}{\sum }h_{e}{\langle \left(\nabla u-m\right)\cdot \nu -\phi \;;\;\left(\nabla v-n\right)\cdot \nu -\psi \rangle }_{e}$$

with v=(v,n,ψ). Here, $E_{\Omega }\left(T\right)$ denotes the set of interior edges (d=2) or faces (d=3) of the elements of the triangulation T of Ω. The expression ${\langle \cdot \;;\;\cdot \rangle }_{e}$ denotes the integral over an edge (or face) e. For elements $e\in T{|}_{\Gamma }$, the vector ν is the outer normal vector on Γ. Moreover, for $e\in E_{\Omega }\left(T\right)$ the bracket ${\left[\cdot \right]}_{e}$ denotes the jump across e and ν is a normal vector of e, i.e., 

$${\left[\left(\nabla u-m\right)\cdot \nu \right]}_{e}≔\left(\nabla u-m\right){|}_{K^{\prime }}\cdot \nu_{K^{\prime }}+\left(\nabla u-m\right){|}_{K^{\prime \prime }}\cdot \nu_{K^{\prime \prime }}$$

on the edge (or face) $e=\bar{K^{\prime }}\cap \bar{K^{\prime \prime }}\in E_{\Omega }\left(T\right)$, which is the intersection of uniquely determined elements $K^{\prime },K^{\prime \prime }\in T$, and $\nu_{K^{\prime }}$ and $\nu_{K^{\prime \prime }}$ denote the exterior normal vectors of $K^{\prime }$ and $K^{\prime \prime }$ respectively. Finally, we denote with $h_{e}$ the diameter of an edge (or face) e. The bilinear form σ is indeed well-defined as is shown as part of the consistency assertion of the following Lemma 4.3.

Lemma 4.3 **Stabilizing Bilinear Form**

*The bilinear form* σ(⋅;⋅) *as defined in*   (4.14)   *is symmetric, positive semi-definite, and consistent, i.e., the exact solution* u=(u,m,ϕ)∈X *satisfies* σ(u;v)=0 *for all* $v\in X_{N}$ *. Moreover, there holds the estimate*

(4.15) $$\underset{q\in H^{1}\left(\Omega \right)\setminus \left\{0\right\}}{\sup}\frac{\left|{\langle m_{N}\;;\;\nabla q\rangle }_{\Omega }\right|}{{\left\|q\right\|}_{H^{1}\left(\Omega \right)}}≲\sigma {\left(0,m_{N},0;0,m_{N},0\right)}^{1/2}\;\text{for all}\;u_{N}=\left(0,m_{N},0\right)\in \underset{N}{\ker}\;b\text{.}$$

Remark 4.4 $m\in L^{2}{\left(\Omega \right)}^{d}$ together with $\sup_{q\in H^{1}\left(\Omega \right)\setminus \left\{0\right\}}\frac{\left|{\langle m\;;\;\nabla q\rangle }_{\Omega }\right|}{{\left\|q\right\|}_{H^{1}\left(\Omega \right)}}=0$ implies

(4.16) $$\mathrm{div}\;\left(m\chi_{\Omega }\right)=0\;\text{in}\;D{\left(R^{d}\right)}^{\prime }\text{,}$$

since for $\varphi \in D\left(R^{d}\right)$ we have ${\langle \mathrm{div}\;\left(m\chi_{\Omega }\right)\;;\;\varphi \rangle }_{R^{d}}=-{\langle m\chi_{\Omega }\;;\;\nabla \varphi \rangle }_{R^{d}}=-{\langle m\;;\;\nabla \varphi \rangle }_{\Omega }$.  ■

Proof of Lemma 4.3 Clearly, σ is a symmetric and positive semi-definite bilinear form. To see that it is well-defined and consistent, it is sufficient to note that by Lemma 4.2 the jump terms and the boundary terms in (4.14) vanish for u=(u,m,ϕ)∈kerb.

To prove the estimate (4.15), we employ the Clément interpolation operator $I_{N}:H^{1}\left(\Omega \right)⟶S^{1,1}\left(T\right)≔\left\{u\in H^{1}\left(\Omega \right)\;:\;\forall K\in T:u{|}_{K}\in P^{1}\left(K\right)\right\}$ of  [23]. For $u_{N}=\left(0,m_{N},0\right)\in \ker_{N}\;b$ we have

$$0=b\left(0,m_{N},0;q,0\right)={\langle m_{N}\;;\;\nabla q\rangle }_{\Omega }\;\text{for all}\;q\in S_{*}^{1,1}\left(\Omega \right)\text{,}$$

and this equation also holds for all $q\in S^{1,1}\left(T\right)$. Observe now for $q\in H^{1}\left(\Omega \right)$

$$|{\langle m_{N}\;;\;\nabla \left(q-I_{N}q\right)\rangle }_{\Omega }|=|\underset{K\in T}{\sum }{\langle m_{N}\;;\;\nabla \left(q-I_{N}q\right)\rangle }_{K}|=|\underset{K\in T}{\sum }{\langle m_{N}\cdot \nu \;;\;q-I_{N}q\rangle }_{\partial K}|=|\underset{e\in E_{\Omega }\left(T\right)}{\sum }{\langle {\left[m_{N}\cdot \nu \right]}_{e}\;;\;q-I_{N}q\rangle }_{e}+\underset{e\in T{|}_{\Gamma }}{\sum }{\langle m_{N}\cdot \nu \;;\;q-I_{N}q\rangle }_{e}|\text{.}$$

Application of standard properties of the Clément interpolant yields the claimed estimate

$$\underset{q\in H^{1}\left(\Omega \right)\setminus \left\{0\right\}}{\sup}\frac{\left|{\langle m_{N}\;;\;\nabla q\rangle }_{\Omega }\right|}{{\left\|q\right\|}_{H^{1}\left(\Omega \right)}}=\underset{q\in H^{1}\left(\Omega \right)\setminus \left\{0\right\}}{\sup}\frac{\left|{\langle m_{N}\;;\;\nabla \left(q-I_{N}q\right)\rangle }_{\Omega }\right|}{{\left\|q\right\|}_{H^{1}\left(\Omega \right)}}≲{\left\{\underset{e\in E_{\Omega }\left(T\right)}{\sum }h_{e}\overset{2}{\underset{e}{\left\|{\left[m_{N}\cdot \nu \right]}_{e}\right\|}}\right\}}^{1/2}+{\left\{\underset{e\in T{|}_{\Gamma }}{\sum }h_{e}\overset{2}{\underset{e}{\left\|m_{N}\cdot \nu \right\|}}\right\}}^{1/2}≲\sigma {\left(0,m_{N},0;0,m_{N},0\right)}^{1/2}\text{.}$$

 □

We formulate now the stabilized discrete saddle point problem $\left(SPP_{\varepsilon ,\sigma }^{N}\right)$.

Problem 4.5 **$SPP_{\varepsilon ,\sigma }^{N}$**

Find $u_{N}=\left(u_{N},m_{N},\phi_{N}\right)\in X_{N}$ and $p_{N}=\left(p_{N},\zeta_{N}\right)\in M_{N}$ such that

(4.17) $$a_{N}^{\sigma }\left(u_{N};v\right)+b\left(v;p_{N}\right)={\langle f\;;\;n\rangle }_{\Omega }\;\text{for all}\;v=\left(v,n,\psi \right)\in X_{N}\text{,}$$

(4.18) $$b\left(u_{N};q\right)=0\;\text{for all}\;q=\left(q,\theta \right)\in M_{N}\text{,}$$

with $a_{N}^{\sigma }\left(u_{N};v\right)≔a_{N}\left(u_{N};v\right)+\sigma \left(u_{N};v\right)$.

The following theorem states existence and uniqueness of the solution $\left(u_{N},m_{N},\phi_{N};p_{N},\zeta_{N}\right)$ of the stabilized discrete saddle point problem.

Theorem 4.6 **Stability and (Unique) Solvability of the Discrete Saddle Point Problem ($SPP_{\varepsilon ,\sigma }^{N}$)**

*The following statements are true:*

1. *The discrete problem* $\left(SPP_{\varepsilon ,\sigma }^{N}\right)$ *has solutions.*
2. *The variables* $u_{N}$ *and* $\phi_{N}$ *as well as the Lagrange multipliers* $p_{N}$ *and* $\zeta_{N}$ *are uniquely determined in* $\left(SPP_{\varepsilon ,\sigma }^{N}\right)$*.*
3. *If* $\varphi^{**}$ *is given as in*Example  1.2   *(“uniaxial case”), the discrete problem* $\left(SPP_{\varepsilon ,\sigma }^{N}\right)$ *is uniquely solvable.*

Proof Existence of solutions $\left(u_{N},m_{N},\phi_{N};p_{N},\zeta_{N}\right)$ for $\left(SPP_{\varepsilon ,\sigma }^{N}\right)$ as well as uniqueness of the variables $u_{N}$ and $\phi_{N}$ and the Lagrange multipliers $p_{N}$ and $\zeta_{N}$ follow as in the continuous case, cf.  Proposition 3.2. Let $\left(u_{N,i};p_{N,i}\right)≔\left(u_{N,i},m_{N,i},\phi_{N,i};p_{N,i},\zeta_{N,i}\right)$, for i=1,2 be two solutions of $\left(SPP_{\varepsilon ,\sigma }^{N}\right)$. We use the abbreviations $e_{u}≔u_{N,2}-u_{N,1}$, $e_{m}≔m_{N,2}-m_{N,1}$, $e_{\phi }≔\phi_{N,2}-\phi_{N,1}$, $e_{p}≔p_{N,2}-p_{N,1}$ and $e_{\zeta }≔\zeta_{N,2}-\zeta_{N,1}$. From (4.18) we obtain

(4.19) $$-{\langle \nabla e_{u}-e_{m}\;;\;\nabla q\rangle }_{\Omega }-{\langle We_{u}+\left(K^{\prime }-1/2\right)e_{\phi }\;;\;q\rangle }_{\Gamma }+{\langle Ve_{\phi }-\left(K-1/2\right)e_{u}\;;\;\theta \rangle }_{\Gamma }=0$$

for all $q=\left(q,\theta \right)\in M_{N}$; hence $\left(e_{u},e_{m},e_{\phi }\right)\in \ker_{N}\;b$. The key step consists in showing $\left(e_{u},e_{m},e_{\phi }\right)\in \ker\;b$, since then the same arguments as in the continuous can be employed to show uniqueness.

Eq. (4.17) with $v≔u_{N,2}-u_{N,1}=\left(e_{u},e_{m},e_{\phi }\right)$ yields together with (4.19)

(4.20) $${\left\|\nabla e_{u}\right\|}_{\Omega }^{2}+{\langle We_{u}\;;\;e_{u}\rangle }_{\Gamma }+\frac{1}{2}{\langle \left(K^{\prime }-1/2\right)e_{\phi }\;;\;e_{u}\rangle }_{\Gamma }+\overset{d-1}{\underset{i=1}{\sum }}{\left\|e_{m}\cdot z_{i}\right\|}_{\Omega }^{2}+{\langle \lambda_{N,2}m_{N,2}-\lambda_{N,1}m_{N,1}\;;\;e_{m}\rangle }_{\Omega }+\frac{1}{2}{\langle \left(K-1/2\right)e_{u}\;;\;e_{\phi }\rangle }_{\Gamma }+\sigma \left(u_{N,2}-u_{N,1};u_{N,2}-u_{N,1}\right)=0\text{.}$$

Eq. (4.19) with $q=\left(0,e_{\phi }\right)$ gives ${\langle Ve_{\phi }-\left(K-1/2\right)e_{u}\;;\;e_{\phi }\rangle }_{\Gamma }=0$, and (4.20) simplifies to

(4.21) $${\left\|\nabla e_{u}\right\|}_{\Omega }^{2}+{\langle We_{u}\;;\;e_{u}\rangle }_{\Gamma }+{\langle Ve_{\phi }\;;\;e_{\phi }\rangle }_{\Gamma }+\overset{d-1}{\underset{i=1}{\sum }}{\left\|e_{m}\cdot z_{i}\right\|}_{\Omega }^{2}+{\langle \lambda_{N,2}m_{N,2}-\lambda_{N,1}m_{N,1}\;;\;e_{m}\rangle }_{\Omega }+\sigma \left(u_{N,2}-u_{N,1};u_{N,2}-u_{N,1}\right)=0\text{.}$$

In  [4, Theorem 3.1], it is shown that $\left(\lambda_{N,2}m_{N,2}-\lambda_{N,1}m_{N,1}\right)\cdot e_{m}\geq 0$ pointwise almost everywhere in Ω. The non-negativity of the bilinear form σ together with the semi-ellipticity of W and the ellipticity of V on $H_{*}^{-1/2}\left(\Gamma \right)$ lead to $e_{u}=0$, $e_{m}\cdot z_{i}=0$, for i=1,…,d−1, and $e_{\phi }=0$. From estimate  (4.15) we have

(4.22) $$\underset{q\in H^{1}\left(\Omega \right)\setminus \left\{0\right\}}{\sup}\frac{\left|{\langle e_{m}\;;\;\nabla q\rangle }_{\Omega }\right|}{{\left\|q\right\|}_{H^{1}\left(\Omega \right)}}≲\sigma {\left(0,e_{m},0;0,e_{m},0\right)}^{1/2}=0\text{,}$$

which implies $\left(e_{u},e_{m},e_{\phi }\right)=\left(0,e_{m},0\right)\in \ker\;b$. Furthermore, we deduce $\mathrm{div}\;\left(e_{m}\chi_{\Omega }\right)=0$ in $D{\left(R^{d}\right)}^{\prime }$ and hence $e_{m}\chi_{\Omega }\in H\left(\mathrm{div};R^{d}\right)$ with $\mathrm{div}\;\left(e_{m}\chi_{\Omega }\right)=0$ in $L^{2}\left(R^{d}\right)$. This observation combined with $e_{m}\cdot z_{i}=0$, for i=1,…,d−1 enables us to prove $e_{m}\chi_{\Omega }=0$ on $R^{d}$ by smoothing techniques as first noted in [20, Satz 2.12]. This yields uniqueness of $m_{N}$. Finally, the discrete inf–sup condition of the bilinear form b ensures uniqueness of the Lagrange multiplier $p_{N}=\left(p_{N},\zeta_{N}\right)$. □

Remark 4.7 The stabilization terms employed here are closely related to the ideas discussed in [24–26]. While the primary concern of these references is to enhance the stability for the Lagrange multiplier, the bilinear form b here is trivially inf–sup stable. The purpose of our term σ is to increase stability for the primal variables (u,m,ϕ).  ■

### 4.4 *A priori* error estimation

In this section, we present a full *a priori* error analysis—in Theorem 4.8 for general functions $\varphi^{**}$ and in Theorem 4.9 for the special case of uniaxial materials given in Example 1.2. In both theorems, the continuous problem is understood to be (SPP) and the discrete problem $\left(SPP_{\varepsilon ,\sigma }^{N}\right)$.

We start in Theorem 4.8 with a general *a priori* estimate for arbitrary anisotropy densities $\varphi^{**}$, which gives convergence $O\left(h^{2}+\varepsilon \right)$ (given sufficient regularity).

Define the seminorm ${\left|\cdot \right|}_{a}$ on X by

(4.23) $${\left|u\right|}_{a}^{2}≔{\left\|\nabla u\right\|}_{\Omega }^{2}+{\left\|u\right\|}_{1/2,\Gamma }^{2}+{\left\|\phi \right\|}_{-1/2,\Gamma }^{2}\text{.}$$

The seminorm ${\left|\cdot \right|}_{\sigma }$ is induced by the symmetric positive semi-definite bilinear form σ of (4.14) in the standard way by

(4.24) $${\left|u\right|}_{\sigma }^{2}≔\sigma \left(u;u\right)\text{.}$$

Theorem 4.8 ***A Priori* Estimate**

*Let* (u;p)=(u,m,ϕ;p,ζ) *and* $\left(u_{N};p_{N}\right)=\left(u_{N},m_{N},\phi_{N};p_{N},\zeta_{N}\right)$ *be solutions of*   Problem  3.1(SPP) *and*   Problem  4.5$\left(SPP_{\varepsilon ,\sigma }^{N}\right)$ *. Fix* $c_{2}$*,* $c_{3}>0$ *. The following *a priori* estimate holds for all* $\left(u_{T};p_{T}\right)=\left(u_{T},m_{T},\phi_{T};p_{T},\zeta_{T}\right)\in X_{N}\times M_{N}$*:*

(4.25) $${\left|u-u_{N}\right|}_{a}^{2}+{\langle \nabla \varphi^{**}\circ m-\nabla \varphi^{**}\circ m_{N}\;;\;m-m_{N}\rangle }_{\Omega }+{\left|u-u_{N}\right|}_{\sigma }^{2}+{\left\|p-p_{N}\right\|}_{M}^{2}\leq C_{\gamma }\left\{{\left\|u-u_{T}\right\|}_{X}^{2}+{\left|u-u_{T}\right|}_{\sigma }^{2}+{\left\|p-p_{T}\right\|}_{\overset{˜}{T}}^{2}+{\left\|p-p_{T}\right\|}_{M}^{2}+{\left\|\varepsilon^{1/2}\lambda_{m}m\right\|}_{\Omega }^{2}-{\left\|\varepsilon^{1/2}\lambda_{N}m_{N}\right\|}_{\Omega }^{2}\right\}+c_{2}{\left\|\nabla \varphi^{**}\circ m-\nabla \varphi^{**}\circ m_{N}\right\|}_{\Omega }^{2}+c_{3}{\left\|\lambda_{m}m-\lambda_{N}m_{N}\right\|}_{\Omega }^{2}\text{.}$$

*The constant* $C_{\gamma }>0$ *depends on the domain* Ω*, the shape regularity of the triangulation* T *. Furthermore, it depends on* $C_{\sigma }>0$ *of*   Lemma  4.12   *and the reciprocals of the arbitrary, chosen* $c_{2}$*,* $c_{3}>0$ *. The mesh-dependent norm* ${\left\|p-p_{T}\right\|}_{\overset{˜}{T}}$ *is defined by*

(4.26) $${\left\|p-p_{T}\right\|}_{\overset{˜}{T}}^{2}≔{\left\|p-p_{T}\right\|}_{T}^{2}+{\left\|p-p_{T}\right\|}_{1/2,\Gamma }^{2}+{\left\|\zeta -\zeta_{T}\right\|}_{-1/2,\Gamma }^{2}≔\underset{e\in E\left(T\right)}{\sum }\overset{-1}{\underset{e}{h}}{\left\|p-p_{T}\right\|}_{e}^{2}+{\left\|p-p_{T}\right\|}_{1/2,\Gamma }^{2}+{\left\|\zeta -\zeta_{T}\right\|}_{-1/2,\Gamma }^{2}\text{.}$$

Given sufficient regularity, the right-hand side of (4.25) is $O\left(h^{2}+\varepsilon \right)$. In the uniaxial case, this upper bound is improved to $O\left(h^{2}+\varepsilon^{2}\right)$ in the following Theorem 4.9. The power of h is optimal for lowest-order elements, and the power of ε is observed to be optimal in numerical studies (cf.  Remark 4.13 ahead).

Theorem 4.9 ***A Priori* Estimate for the Uniaxial Case**

*Assume in addition to the assumptions of*   Theorem  4.8   *that*

(4.27) $$C_{0}{\left\|\nabla \varphi^{**}\circ m_{1}-\nabla \varphi^{**}\circ m_{2}\right\|}_{\Omega }^{2}\leq {\langle \nabla \varphi^{**}\circ m_{1}-\nabla \varphi^{**}\circ m_{2}\;;\;m_{1}-m_{2}\rangle }_{\Omega }\text{.}$$

*Then there holds the *a priori* estimate*

(4.28) $${\left|u-u_{N}\right|}_{a}^{2}+{\left\|\nabla \varphi^{**}\circ m-\nabla \varphi^{**}\circ m_{N}\right\|}_{\Omega }^{2}+{\left\|\lambda_{m}m-\lambda_{N}m_{N}\right\|}_{\Omega }^{2}+{\left|u-u_{N}\right|}_{\sigma }^{2}+{\left\|p-p_{N}\right\|}_{M}^{2}\leq \left(C_{1}+C_{2}{\left\|\varepsilon \right\|}_{L^{\infty }\left(\Omega \right)}\right)\left\{{\left\|u-u_{T}\right\|}_{X}^{2}+{\left|u-u_{T}\right|}_{\sigma }^{2}+{\left\|p-p_{T}\right\|}_{\overset{˜}{T}}^{2}+{\left\|p-p_{T}\right\|}_{M}^{2}+{\left\|\lambda_{m}m-\Pi \left(\lambda_{m}m\right)\right\|}_{\Omega }^{2}\right\}+C_{3}{\left\|\varepsilon \right\|}_{L^{\infty }\left(\Omega \right)}{\left\|\varepsilon^{1/2}\lambda_{m}m\right\|}_{\Omega }^{2}\text{,}$$

*where* $\Pi :L^{2}{\left(\Omega \right)}^{d}↠S^{0,0}{\left(T\right)}^{d}$ *denotes the* $L^{2}{\left(\Omega \right)}^{d}$*-orthogonal projection. The constants* $C_{1}$*,* $C_{2}$*,* $C_{3}>0$ *depend only on* $C_{0}$*, the domain* Ω*, the shape regularity of the triangulation* T*, and on* $C_{\sigma }>0$ *of*   Lemma  4.12*.*

Corollary 4.10 *In addition to the hypotheses of*   Theorems  4.8 and 4.9*, assume for the solution* $\left(u,m,\phi ,\lambda_{m},p,\zeta \right)$ *of problem* (SPP)*the regularity assertions* $u,p\in H^{2}\left(\Omega \right)\cap H_{*}^{1}\left(\Omega \right)$*,* $m\in H^{1}{\left(\Omega \right)}^{d}$*,* $\lambda_{m}m\in H^{1}{\left(\Omega \right)}^{d}$ *and* $\phi ,\zeta \in H_{*}^{1/2}\left(\Gamma \right)$ *. Then, with* $h≔\max_{K\in T}h_{K}$*, there holds*

(4.29) $${\left|u-u_{N}\right|}_{a}+{\left\|\nabla \varphi^{**}\circ m-\nabla \varphi^{**}\circ m_{N}\right\|}_{\Omega }+{\left\|\lambda_{m}m-\lambda_{N}m_{N}\right\|}_{\Omega }+{\left|u-u_{N}\right|}_{\sigma }+{\left\|p-p_{N}\right\|}_{M}=O\left(h+{\left\|\varepsilon \right\|}_{L^{\infty }\left(\Omega \right)}\right)\text{.}$$

Proof The result follows from (4.28) with the choices $u_{T}=I_{*,\Gamma }u$, $p_{T}=I_{*,\Gamma }p$, $m_{T}=\Pi m$, $\phi_{T}=Q\phi$, and $\zeta_{T}=Q\zeta$. Here, $Q:H_{*}^{1/2}\left(\Gamma \right)↠S_{*}^{0,0}\left(\tau \right)$ denotes the usual $L^{2}$-orthogonal projection. The operator $I_{*,\Gamma }:H_{*}^{1}\left(\Omega \right)↠S_{*}^{1,1}\left(T\right)$ is a quasi interpolation operator, which can be constructed with techniques introduced in  [27]. For example, letting $I^{SZ}:H^{1}\left(\Omega \right)↠S^{1,1}\left(T\right)$ be the Scott–Zhang operator and $N_{\Gamma }$ be the nodes of the triangulation on Γ with corresponding hat functions $\varphi_{z}$, one can set

$$I_{*,\Gamma }u≔I^{SZ}u-\underset{z\in N_{\Gamma }}{\sum }\varphi_{z}\frac{{\langle u-I^{SZ}u;\varphi_{z}\rangle }_{\Gamma }}{{\langle \varphi_{z};1\rangle }_{\Gamma }}\text{.}$$

Since the functions ${\left(\varphi_{z}\right)}_{z\in N_{\Gamma }}$ form a partition of unity on Γ, this operator has the desired mapping property $I_{*,\Gamma }:H_{*}^{1}\left(\Omega \right)\to S_{*}^{1,1}\left(T\right)$. The local approximation properties of $I_{*,\Gamma }$ follow from the local approximation properties of $I^{SZ}$. We refer to [22] for an alternative construction with tighter locality. □

We start by formulating the Galerkin orthogonalities available to us: Subtracting (4.17) from (3.1) and (4.18) from (3.2) yields together with the consistency of σ the two relations

(4.30) $$a_{bl}\left(u-u_{N};v_{N}\right)+{\langle \nabla \varphi^{**}\circ m-\nabla \varphi^{**}\circ m_{N}\;;\;n_{N}\rangle }_{\Omega }+{\langle \lambda_{m}m-\lambda_{N}m_{N}\;;\;n_{N}\rangle }_{\Omega }+\sigma \left(u-u_{N};v_{N}\right)+b\left(v_{N};p-p_{N}\right)=0\;\text{for all}\;v_{N}=\left(v_{N},n_{N},\psi_{N}\right)\in X_{N}\text{,}$$

and

(4.31) $$b\left(u-u_{N};q_{N}\right)=0\;\text{for all}\;q_{N}=\left(q_{N},\theta_{N}\right)\in M_{N}\text{,}$$

where we set

(4.32) $$a_{bl}\left(u;v\right)≔{\langle \nabla u\;;\;\nabla v\rangle }_{\Omega }+{\langle Wu+1/2\left(K^{\prime }-1/2\right)\phi \;;\;v\rangle }_{\Gamma }+\frac{1}{2}{\langle \left(K-1/2\right)u\;;\;\psi \rangle }_{\Gamma }\text{.}$$

We have the following estimates.

Lemma 4.11 *With the definition of* $a_{bl}\left(\cdot ;\cdot \right)$ *in*   (4.32)   *there holds*

(4.33) $$\left|a_{bl}\left(u;v\right)\right|\leq C_{a}{\left|u\right|}_{a}{\left|v\right|}_{a}\;\text{for all}\;u\in X\text{.}$$

*If* u∈kerb *or* $u\in \ker_{N}\;b$*, then*

(4.34) $$a_{bl}\left(u;u\right)≃{\left|u\right|}_{a}^{2}\text{.}$$

*Furthermore, there holds*

(4.35) $${\left|u\right|}_{a}\leq C_{a,X}{\left\|u\right\|}_{X}\;\text{for all}\;u\in X\text{.}$$

Proof Estimates (4.33) and (4.35) are straightforward. We show (4.34). From u∈kerb or $u\in \ker_{N}\;b$, we get ${\langle \left(K-1/2\right)u\;;\;\phi \rangle }_{\Gamma }={\langle V\phi \;;\;\phi \rangle }_{\Gamma }$. The ellipticity of W on $H_{*}^{1/2}\left(\Gamma \right)$ and of V on $H_{*}^{-1/2}\left(\Gamma \right)$ now yields

$$a_{bl}\left(u;u\right)={\left\|\nabla u\right\|}_{\Omega }^{2}+{\langle Wu\;;\;u\rangle }_{\Gamma }+{\langle \left(K-1/2\right)u\;;\;\phi \rangle }_{\Gamma }={\left\|\nabla u\right\|}_{\Omega }^{2}+{\langle Wu\;;\;u\rangle }_{\Gamma }+{\langle V\phi \;;\;\phi \rangle }_{\Gamma }≳{\left\|\nabla u\right\|}_{\Omega }^{2}+{\left\|u\right\|}_{1/2,\Gamma }^{2}+{\left\|\phi \right\|}_{-1/2,\Gamma }^{2}\text{.}$$

 □

Lemma 4.12 *There exists* $C_{\sigma }>0$ *depending only on the shape regularity of* T *such that*

(4.36) $$\left|\sigma \left(u;v\right)\right|\leq {\left|u\right|}_{\sigma }{\left|v\right|}_{\sigma }\text{.}\;\forall u,v\in X_{N}+\ker b\text{,}$$

(4.37) $${\left|u_{N}\right|}_{\sigma }\leq C_{\sigma }{\left\|u_{N}\right\|}_{X}\;\forall u_{N}\in X_{N}\text{.}$$

Proof (4.36) is again straightforward. We prove (4.37).

(4.38) $${\left|u_{N}\right|}_{\sigma }^{2}=\underset{e\in E_{\Omega }\left(T\right)}{\sum }h_{e}\overset{2}{\underset{e}{\left\|{\left[\left(\nabla u_{N}-m_{N}\right)\cdot \nu \right]}_{e}\right\|}}+\underset{e\in T{|}_{\Gamma }}{\sum }h_{e}\overset{2}{\underset{e}{\left\|\left(\nabla u_{N}-m_{N}\right)\cdot \nu -\phi_{N}\right\|}}=\underset{e\in E_{\Omega }\left(T\right)}{\sum }h_{e}\overset{2}{\underset{e}{\left\|{\left[\left(\nabla u_{N}-m_{N}\right)\cdot \nu \right]}_{e}\right\|}}+2\underset{e\in T{|}_{\Gamma }}{\sum }h_{e}\overset{2}{\underset{e}{\left\|\left(\nabla u_{N}-m_{N}\right)\cdot \nu \right\|}}+2\underset{e\in T{|}_{\Gamma }}{\sum }h_{e}\overset{2}{\underset{e}{\left\|\phi_{N}\right\|}}\text{.}$$

To estimate the first two sums we use a transformation to the reference element and norm equivalence on finite dimensional spaces on the reference element. This yields

(4.39) $$\begin{array}{cc} & \underset{e\in E_{\Omega }\left(T\right)}{\sum }h_{e}\overset{2}{\underset{e}{\left\|{\left[\left(\nabla u_{N}-m_{N}\right)\cdot \nu \right]}_{e}\right\|}}+2\underset{e\in T{|}_{\Gamma }}{\sum }h_{e}\overset{2}{\underset{e}{\left\|\left(\nabla u_{N}-m_{N}\right)\cdot \nu \right\|}}\leq {\overset{˜}{C}}_{\sigma }^{2}{\left\|\nabla u_{N}-m_{N}\right\|}_{\Omega }^{2}\text{.} \end{array}$$

The last term in the sum (4.38) is estimated as $h_{e}{\left\|\phi_{N}\right\|}_{e}≲{\left\|\phi_{N}\right\|}_{H^{-1/2}\left(\Gamma \right)}$ by an inverse estimate (cf. [28, Thm. 3.5], [29, Thm. 4.6], [30, Thm. 3.6]). Together with (4.39) this yields

(4.40) $${\left|u_{N}\right|}_{\sigma }^{2}\leq C_{\sigma }^{2}\left({\left\|\nabla u_{N}\right\|}_{\Omega }^{2}+{\left\|m_{N}\right\|}_{\Omega }^{2}+{\left\|\phi_{N}\right\|}_{H^{-1/2}\left(\Gamma \right)}^{2}\right)=C_{\sigma }^{2}{\left\|u_{N}\right\|}_{X}^{2}\text{.}$$

 □

In the proofs of Theorems 4.8 and 4.9 we will use the following abbreviations:

(4.41) $$d≔\nabla \varphi^{**}\circ m\text{,}\;d_{N}≔\nabla \varphi^{**}\circ m_{N}\text{,}$$

(4.42) $$\ell :=\lambda_{m}m\text{,}\;\ell_{N}≔\lambda_{N}m_{N}\text{.}$$

Moreover, we denote with lower case letters constants that can be chosen arbitrarily small, whereas upper case letters denote constants that are independent of mesh parameters but depend on the chosen lower case constants.

Proof of Theorem 4.8 The proof follows an often employed path in saddle point theory. First, a best approximation result is obtained in the constrained space $\ker_{N}\;b$. This is done in Steps 1–7. In the final Step 8, this restriction is lifted.

In Steps 1–7, we consider $u_{T}^{⋆}=\left(u_{T}^{⋆},m_{T}^{⋆},\phi_{T}^{⋆}\right)\in \ker_{N}\;b\subset X_{N}$ and define $d_{T}^{⋆}≔\nabla \varphi^{**}\circ m_{T}^{⋆}$.

***Step* 1:***Claim:* There exists $0<C_{1}\leq 1$ such that

(4.43) $$S_{1}≔C_{1}{\left|u_{T}^{⋆}-u_{N}\right|}_{a}^{2}+{\langle d_{T}^{⋆}-d_{N}\;;\;m_{T}^{⋆}-m_{N}\rangle }_{\Omega }+{\langle \ell -\ell_{N}\;;\;m-m_{N}\rangle }_{\Omega }+\sigma \left(u_{T}^{⋆}-u_{N};u_{T}^{⋆}-u_{N}\right)\leq a_{bl}\left(u_{T}^{⋆}-u;u_{T}^{⋆}-u_{N}\right)+{\langle d_{T}^{⋆}-d\;;\;m_{T}^{⋆}-m_{N}\rangle }_{\Omega }+{\langle \ell -\ell_{N}\;;\;m-m_{T}^{⋆}\rangle }_{\Omega }+\sigma \left(u_{T}^{⋆}-u;u_{T}^{⋆}-u_{N}\right)-b\left(u_{T}^{⋆}-u_{N};p-p_{N}\right)\text{.}$$

Indeed, since $u_{T}^{⋆}-u_{N}\in \ker_{N}\;b$ the proof of Lemma 4.11 showed

(4.44) $$S_{1}\leq a_{bl}\left(u_{T}^{⋆}-u;u_{T}^{⋆}-u_{N}\right)+a_{bl}\left(u-u_{N};u_{T}^{⋆}-u_{N}\right)+{\langle d_{T}^{⋆}-d\;;\;m_{T}^{⋆}-m_{N}\rangle }_{\Omega }+{\langle d-d_{N}\;;\;m_{T}^{⋆}-m_{N}\rangle }_{\Omega }+{\langle \ell -\ell_{N}\;;\;m-m_{T}^{⋆}\rangle }_{\Omega }+{\langle \ell -\ell_{N}\;;\;m_{T}^{⋆}-m_{N}\rangle }_{\Omega }+\sigma \left(u_{T}^{⋆}-u;u_{T}^{⋆}-u_{N}\right)+\sigma \left(u-u_{N};u_{T}^{⋆}-u_{N}\right)\text{.}$$

The Galerkin orthogonality (4.30) with $v_{N}=u_{T}^{⋆}-u_{N}$ then proves (4.43).

***Step* 2:***Claim:* For arbitrary $p_{T}\in M_{N}$ and arbitrary $c_{Y,1}>0$, the last term in (4.43) can be estimated as follows:

(4.45) $$\left|b\left(u_{T}^{⋆}-u_{N};p-p_{N}\right)\right|\leq C_{a,b}\left\{c_{Y,1}\left\{\sigma \left(u_{T}^{⋆}-u_{N};u_{T}^{⋆}-u_{N}\right)+a_{bl}\left(u_{T}^{⋆}-u_{N};u_{T}^{⋆}-u_{N}\right)\right\}+\frac{1}{c_{Y,1}}{\left\|p-p_{T}\right\|}_{\overset{˜}{T}}^{2}\right\}\text{.}$$

To see this, observe that $u_{T}^{⋆},u_{N}\in \ker_{N}\;b$ implies

(4.46) $$b\left(u_{T}^{⋆}-u_{N};p-p_{N}\right)=b\left(u_{T}^{⋆}-u_{N};p-p_{T}\right)+\underset{=0}{\underset{︸}{b\left(u_{T}^{⋆}-u_{N};p_{T}-p_{N}\right)}}\text{.}$$

In order to estimate $b\left(u_{T}^{⋆}-u_{N};p-p_{T}\right)$, let $v_{N}≔u_{T}^{⋆}-u_{N}\in \ker_{N}\;b\subset X_{N}$ and $q≔p-p_{T}\in M$. Then

(4.47) $$\left|b\left(v_{N};q\right)\right|\leq \left|{\langle \nabla v_{N}-n_{N}\;;\;\nabla q\rangle }_{\Omega }\right|+\left|{\langle Wv_{N}+\left(K^{\prime }-1/2\right)\psi_{N}\;;\;q\rangle }_{\Gamma }\right|+\left|{\langle V\psi_{N}-\left(K-1/2\right)v_{N}\;;\;\theta \rangle }_{\Gamma }\right|\leq \left|{\langle \nabla v_{N}-n_{N}\;;\;\nabla q\rangle }_{\Omega }\right|+{\left\|Wv_{N}\right\|}_{-1/2,\Gamma }{\left\|q\right\|}_{1/2,\Gamma }+{\left\|\left(K^{\prime }-1/2\right)\psi_{N}\right\|}_{-1/2,\Gamma }{\left\|q\right\|}_{1/2,\Gamma }+{\left\|V\psi_{N}\right\|}_{1/2,\Gamma }{\left\|\theta \right\|}_{-1/2,\Gamma }+{\left\|\left(K-1/2\right)v_{N}\right\|}_{1/2,\Gamma }{\left\|\theta \right\|}_{-1/2,\Gamma }\text{.}$$

We next introduce the bilinear form σ by integrating by parts in the first term:

(4.48) $$\left|{\langle \nabla v_{N}-n_{N}\;;\;\nabla q\rangle }_{\Omega }\right|=|\underset{K\in T}{\sum }{\langle \nabla v_{N}-n_{N}\;;\;\nabla q\rangle }_{K}|=|\underset{e\in E_{\Omega }\left(T\right)}{\sum }{\langle {\left[\left(\nabla v_{N}-n_{N}\right)\cdot \nu \right]}_{e}\;;\;q\rangle }_{e}+\underset{e\in E_{\Gamma }\left(T\right)}{\sum }\left\{{\langle \left(\nabla v_{N}-n_{N}\right)\cdot \nu -\psi_{N}\;;\;q\rangle }_{e}+{\langle \psi_{N}\;;\;q\rangle }_{e}\right\}|\leq \left[{\left\{\underset{e\in E_{\Omega }\left(T\right)}{\sum }h_{e}\overset{2}{\underset{e}{\left\|{\left[\left(\nabla v_{N}-n_{N}\right)\cdot \nu \right]}_{e}\right\|}}\right\}}^{1/2}+{\left\{\underset{e\in E_{\Gamma }\left(T\right)}{\sum }h_{e}\overset{2}{\underset{e}{\left\|\left(\nabla v_{N}-n_{N}\right)\cdot \nu -\psi_{N}\right\|}}\right\}}^{1/2}\right]{\left\{\underset{e\in E\left(T\right)}{\sum }\overset{-1}{\underset{e}{h}}\overset{2}{\underset{e}{\left\|q\right\|}}\right\}}^{1/2}+{\left\|\psi_{N}\right\|}_{-1/2,\Gamma }{\left\|q\right\|}_{1/2,\Gamma }\leq 2^{1/2}\sigma {\left(v_{N};v_{N}\right)}^{1/2}\underset{=Tq}{\underset{︸}{{\left\{\underset{e\in E\left(T\right)}{\sum }\overset{-1}{\underset{e}{h}}\overset{2}{\underset{e}{\left\|q\right\|}}\right\}}^{1/2}}}+{\left\|\psi_{N}\right\|}_{-1/2,\Gamma }{\left\|q\right\|}_{1/2,\Gamma }=2^{1/2}\sigma {\left(v_{N};v_{N}\right)}^{1/2}{\left\|q\right\|}_{T}+{\left\|\psi_{N}\right\|}_{-1/2,\Gamma }{\left\|q\right\|}_{1/2,\Gamma }\text{.}$$

Substituting into (4.47) gives together with Lemma 4.11 the claimed estimate, namely,

(4.49) $$\left|b\left(v_{N};q\right)\right|\leq C_{b}\left\{\sigma {\left(v_{N};v_{N}\right)}^{1/2}{\left\|q\right\|}_{T}+{\left\|\psi_{N}\right\|}_{-1/2,\Gamma }{\left\|q\right\|}_{1/2,\Gamma }+{\left\|v_{N}\right\|}_{1/2,\Gamma }{\left\|q\right\|}_{1/2,\Gamma }+{\left\|\psi_{N}\right\|}_{-1/2,\Gamma }{\left\|q\right\|}_{1/2,\Gamma }+{\left\|\psi_{N}\right\|}_{-1/2,\Gamma }{\left\|\theta \right\|}_{-1/2,\Gamma }+{\left\|v_{N}\right\|}_{1/2,\Gamma }{\left\|\theta \right\|}_{-1/2,\Gamma }\right\}\leq C_{b}\left[c_{Y,1}\left\{\sigma \left(v_{N};v_{N}\right)+{\left\|\psi_{N}\right\|}_{-1/2,\Gamma }^{2}+{\left\|v_{N}\right\|}_{1/2,\Gamma }^{2}\right\}+\frac{1}{c_{Y,1}}\left\{\underset{={\left\|q\right\|}_{\overset{˜}{T}}^{2}}{\underset{︸}{{\left\|q\right\|}_{T}^{2}+{\left\|q\right\|}_{1/2,\Gamma }^{2}+{\left\|\theta \right\|}_{-1/2,\Gamma }^{2}}}\right\}\right]\leq C_{a,b}\left\{c_{Y,1}\left[\sigma \left(v_{N};v_{N}\right)+a_{bl}\left(v_{N};v_{N}\right)\right\}+\frac{1}{c_{Y,1}}\overset{2}{\underset{\overset{˜}{T}}{\left\|q\right\|}}\right]\text{.}$$

***Step* 3:***Claim:* With constants $C_{2}$, $C_{3}$, $C_{4}$, $C_{5}$, $C_{6}$ arising from Young inequalities there holds:

(4.50) $$C_{2}{\left|u_{T}^{⋆}-u_{N}\right|}_{a}^{2}+{\langle d_{T}^{⋆}-d_{N}\;;\;m_{T}^{⋆}-m_{N}\rangle }_{\Omega }+C_{3}{\left|u_{T}^{⋆}-u_{N}\right|}_{\sigma }^{2}\leq C_{4}{\left|u_{T}^{⋆}-u\right|}_{a}^{2}+{\langle d_{T}^{⋆}-d\;;\;m_{T}^{⋆}-m_{N}\rangle }_{\Omega }+{\langle \ell -\ell_{N}\;;\;m-m_{T}^{⋆}\rangle }_{\Omega }+C_{5}{\left|u_{T}^{⋆}-u\right|}_{\sigma }^{2}+C_{6}{\left\|p-p_{T}\right\|}_{\overset{˜}{T}}^{2}+\frac{1}{2}{\left\|\varepsilon^{1/2}\ell \right\|}_{L^{2}\left(\Omega \right)}^{2}-\frac{1}{2}{\left\|\varepsilon^{1/2}\ell_{N}\right\|}_{L^{2}\left(\Omega \right)}^{2}\text{.}$$

From Steps 1 and 2 we have

(4.51) $$S_{3}≔C_{1}{\left|u_{T}^{⋆}-u_{N}\right|}_{a}^{2}+{\langle d_{T}^{⋆}-d_{N}\;;\;m_{T}^{⋆}-m_{N}\rangle }_{\Omega }+{\langle \ell -\ell_{N}\;;\;m-m_{N}\rangle }_{\Omega }+{\left|u_{T}^{⋆}-u_{N}\right|}_{\sigma }^{2}\leq a_{bl}\left(u_{T}^{⋆}-u;u_{T}^{⋆}-u_{N}\right)+{\langle d_{T}^{⋆}-d\;;\;m_{T}^{⋆}-m_{N}\rangle }_{\Omega }+{\langle \ell -\ell_{N}\;;\;m-m_{T}^{⋆}\rangle }_{\Omega }+\sigma \left(u_{T}^{⋆}-u;u_{T}^{⋆}-u_{N}\right)+C_{a,b}\left[c_{Y,1}\left\{\sigma \left(u_{T}^{⋆}-u_{N};u_{T}^{⋆}-u_{N}\right)+a_{bl}\left(u_{T}^{⋆}-u_{N};u_{T}^{⋆}-u_{N}\right)\right\}+\frac{1}{c_{Y,1}}{\left\|p-p_{T}\right\|}_{\overset{˜}{T}}^{2}\right]\text{.}$$

With Lemmas 4.11–4.12 and the Young inequality, we get

(4.52) $$\begin{array}{cc} a_{bl}\left(u_{T}^{⋆}-u;u_{T}^{⋆}-u_{N}\right) & \leq C_{a}{\left|u_{T}^{⋆}-u\right|}_{a}{\left|u_{T}^{⋆}-u_{N}\right|}_{a}\leq C_{a}\left\{\frac{1}{2c_{Y,2}}{\left|u_{T}^{⋆}-u\right|}_{a}^{2}+\frac{c_{Y,2}}{2}{\left|u_{T}^{⋆}-u_{N}\right|}_{a}^{2}\right\}\text{,}\\ a_{bl}\left(u_{T}^{⋆}-u_{N};u_{T}^{⋆}-u_{N}\right) & \leq C_{a}{\left|u_{T}^{⋆}-u_{N}\right|}_{a}^{2}\text{,}\\ \sigma \left(u_{T}^{⋆}-u;u_{T}^{⋆}-u_{N}\right) & \leq {\left|u_{T}^{⋆}-u\right|}_{\sigma }{\left|u_{T}^{⋆}-u_{N}\right|}_{\sigma }\leq \frac{1}{2c_{Y,3}}{\left|u_{T}^{⋆}-u\right|}_{\sigma }^{2}+\frac{c_{Y,3}}{2}{\left|u_{T}^{⋆}-u_{N}\right|}_{\sigma }^{2}\text{,}\\ \sigma \left(u_{T}^{⋆}-u_{N};u_{T}^{⋆}-u_{N}\right) & \leq {\left|u_{T}^{⋆}-u_{N}\right|}_{\sigma }^{2}\text{,} \end{array}$$

which leads to

(4.53) $$S_{3}\leq \frac{C_{a}}{2c_{Y,2}}{\left|u_{T}^{⋆}-u\right|}_{a}^{2}+\frac{C_{a}c_{Y,2}}{2}{\left|u_{T}^{⋆}-u_{N}\right|}_{a}^{2}+{\langle d_{T}^{⋆}-d\;;\;m_{T}^{⋆}-m_{N}\rangle }_{\Omega }+{\langle \ell -\ell_{N}\;;\;m-m_{T}^{⋆}\rangle }_{\Omega }+\frac{1}{2c_{Y,3}}{\left|u_{T}^{⋆}-u\right|}_{\sigma }^{2}+\frac{c_{Y,3}}{2}{\left|u_{T}^{⋆}-u_{N}\right|}_{\sigma }^{2}+C_{a,b}\left[c_{Y,1}\left\{{\left|u_{T}^{⋆}-u_{N}\right|}_{\sigma }^{2}+C_{a}{\left|u_{T}^{⋆}-u_{N}\right|}_{a}^{2}\right\}+\frac{1}{c_{Y,1}}{\left\|p-p_{T}\right\|}_{\overset{˜}{T}}^{2}\right]\text{.}$$

We use the bound

(4.54) $$\frac{1}{2}{\left\|\varepsilon^{1/2}\ell_{N}\right\|}_{L^{2}\left(\Omega \right)}^{2}-\frac{1}{2}{\left\|\varepsilon^{1/2}\ell \right\|}_{L^{2}\left(\Omega \right)}^{2}\leq {\langle \ell -\ell_{N}\;;\;m-m_{N}\rangle }_{L^{2}\left(\Omega \right)}\text{,}$$

of  [4, Proof of Thm 4.3] and absorb the terms ${\left|u_{T}^{⋆}-u_{N}\right|}_{a}^{2}$ and ${\left|u_{T}^{⋆}-u_{N}\right|}_{\sigma }^{2}$ of the right-hand side of (4.53) in the corresponding terms in $S_{3}$ by taking $c_{Y,1}$, $c_{Y,3}$ sufficiently small. This yields (4.50).

***Step* 4:***Claim:* For any function $u_{T}\in S_{*}^{1,1}\left(T\right)$ there holds the estimate

(4.55) $$S_{4}≔\frac{C_{2}}{2}{\left|u-u_{N}\right|}_{a}^{2}+{\langle d-d_{N}\;;\;m-m_{N}\rangle }_{\Omega }+C_{3}{\left|u_{T}^{⋆}-u_{N}\right|}_{\sigma }^{2}\leq C_{7}{\left|u_{T}^{⋆}-u\right|}_{a}^{2}+{\langle d-d_{N}\;;\;m-m_{T}^{⋆}\rangle }_{\Omega }+{\langle \ell -\ell_{N}\;;\;m-m_{T}^{⋆}\rangle }_{\Omega }+C_{5}{\left|u_{T}^{⋆}-u\right|}_{\sigma }^{2}+C_{6}{\left\|p-p_{T}\right\|}_{\overset{˜}{T}}^{2}+\frac{1}{2}{\left\|\varepsilon^{1/2}\ell \right\|}_{L^{2}\left(\Omega \right)}^{2}-\frac{1}{2}{\left\|\varepsilon^{1/2}\ell_{N}\right\|}_{L^{2}\left(\Omega \right)}^{2}\text{.}$$

First, a triangle inequality and a Young inequality give

(4.56) $$\frac{C_{2}}{2}{\left|u-u_{N}\right|}_{a}^{2}\leq C_{2}{\left|u-u_{T}^{⋆}\right|}_{a}^{2}+C_{2}{\left|u_{T}^{⋆}-u_{N}\right|}_{a}^{2}\text{.}$$

Second, we have the identity

(4.57) $${\langle d-d_{N}\;;\;m-m_{N}\rangle }_{\Omega }={\langle d-d_{N}\;;\;m-m_{T}^{⋆}\rangle }_{\Omega }+{\langle d-d_{T}^{⋆}\;;\;m_{T}^{⋆}-m_{N}\rangle }_{\Omega }+{\langle d_{T}^{⋆}-d_{N}\;;\;m_{T}^{⋆}-m_{N}\rangle }_{\Omega }\text{.}$$

Using these two expressions, we get together with (4.50) the claimed estimate (4.55).

***Step* 5:** For arbitrary $c_{9}$, $c_{10}>0$, the Young inequality proves

(4.58) $$\min\left\{1,\frac{C_{2}}{2},C_{3}\right\}S_{4}\leq \overset{2}{\underset{a}{\left|u-u_{N}\right|}}+{\langle d-d_{N}\;;\;m-m_{N}\rangle }_{\Omega }+{\left|u_{T}^{⋆}-u_{N}\right|}_{\sigma }^{2}\leq C_{8}\left\{{\left|u_{T}^{⋆}-u\right|}_{a}^{2}+{\left\|m-m_{T}^{⋆}\right\|}_{\Omega }^{2}+{\left|u_{T}^{⋆}-u\right|}_{\sigma }^{2}+{\left\|p-p_{T}\right\|}_{\overset{˜}{T}}^{2}\right\}+c_{9}{\left\|d-d_{N}\right\|}_{\Omega }^{2}+c_{10}{\left\|\ell -\ell_{N}\right\|}_{\Omega }^{2}+C_{11}\left\{{\left\|\varepsilon^{1/2}\ell \right\|}_{L^{2}\left(\Omega \right)}^{2}-{\left\|\varepsilon^{1/2}\ell_{N}\right\|}_{L^{2}\left(\Omega \right)}^{2}\right\}\text{.}$$

***Step* 6:** With  (4.58), the triangle inequality proves

(4.59) $${\left|u-u_{N}\right|}_{a}^{2}+{\langle d-d_{N}\;;\;m-m_{N}\rangle }_{\Omega }+{\left|u-u_{N}\right|}_{\sigma }^{2}\leq 2C_{8}\left\{{\left|u_{T}^{⋆}-u\right|}_{a}^{2}+{\left\|m-m_{T}^{⋆}\right\|}_{\Omega }^{2}+{\left\|p-p_{T}\right\|}_{\overset{˜}{T}}^{2}\right\}+2\left(C_{8}+1\right){\left|u_{T}^{⋆}-u\right|}_{\sigma }^{2}+2c_{9}{\left\|d-d_{N}\right\|}_{\Omega }^{2}+2c_{10}{\left\|\ell -\ell_{N}\right\|}_{\Omega }^{2}+2C_{11}\left\{{\left\|\varepsilon^{1/2}\ell \right\|}_{L^{2}\left(\Omega \right)}^{2}-{\left\|\varepsilon^{1/2}\ell_{N}\right\|}_{L^{2}\left(\Omega \right)}^{2}\right\}\text{.}$$

***Step* 7:** In this step we estimate $p-p_{N}$. The proof of the inf–sup condition for the bilinear form b (cf.  (3.12)) shows for arbitrary $q_{N}=\left(q_{N},\theta_{N}\right)\in M_{N}$ the validity of

(4.60) $$\frac{b\left(v_{N};q_{N}\right)}{{\left\|v_{N}\right\|}_{X}}\geq \beta {\left\|q_{N}\right\|}_{M}$$

for positive $\beta =\min\left\{1,c_{1}^{V}\right\}$, if one sets $v_{N}=\left(-q_{N},0,\theta_{N}\right)$. Inserting $p_{N}-q_{N}=\left(p_{N}-q_{N},\zeta_{N}-\theta_{N}\right)$ in place of $q_{N}$ in (4.60) and letting $q_{N}$ still be arbitrary shows with $v_{N}=\left(-\left(p_{N}-q_{N}\right),0,\zeta_{N}-\theta_{N}\right)$

(4.61) $$\frac{b\left(v_{N};p_{N}-q_{N}\right)}{{\left\|v_{N}\right\|}_{X}}\geq \beta {\left\|p_{N}-q_{N}\right\|}_{M}\text{.}$$

Next we split the bilinear form b into two terms and set $q_{N}=p_{T}$, that is,

(4.62) $$\beta {\left\|p_{N}-p_{T}\right\|}_{M}\leq \frac{b\left(v_{N};p_{N}-p_{T}\right)}{{\left\|v_{N}\right\|}_{X}}=\frac{b\left(v_{N};p_{N}-p\right)+b\left(v_{N};p-p_{T}\right)}{{\left\|v_{N}\right\|}_{X}}\text{.}$$

Note that $n_{N}=0$ in the second component of $v_{N}$. The Galerkin orthogonality (4.30) then yields

(4.63) $$b\left(v_{N};p_{N}-p\right)=a_{bl}\left(u-u_{N};v_{N}\right)+\sigma \left(u-u_{N};v_{N}\right)$$

and therefore

(4.64) $$\beta {\left\|p_{N}-p_{T}\right\|}_{M}\leq \frac{a_{bl}\left(u-u_{N};v_{N}\right)+\sigma \left(u-u_{N};v_{N}\right)+b\left(v_{N};p-p_{T}\right)}{{\left\|v_{N}\right\|}_{X}}\leq \frac{C_{a}{\left|u-u_{N}\right|}_{a}{\left|v_{N}\right|}_{a}+{\left|u-u_{N}\right|}_{\sigma }{\left|v_{N}\right|}_{\sigma }+C_{b,2}{\left\|p-p_{T}\right\|}_{M}{\left\|v_{N}\right\|}_{X}}{{\left\|v_{N}\right\|}_{X}}\text{.}$$

Due to Lemmas 4.11 and 4.12 we estimate further with $\overset{˜}{C}=\max\left\{C_{a}C_{a,X},C_{\sigma },C_{b,2}\right\}/\beta$

(4.65) $${\left\|p_{N}-p_{T}\right\|}_{M}\leq \overset{˜}{C}\frac{{\left|u-u_{N}\right|}_{a}{\left\|v_{N}\right\|}_{X}+{\left|u-u_{N}\right|}_{\sigma }{\left\|v_{N}\right\|}_{X}+{\left\|p-p_{T}\right\|}_{M}{\left\|v_{N}\right\|}_{X}}{{\left\|v_{N}\right\|}_{X}}$$

(4.66) $$\leq C\left\{{\left|u-u_{N}\right|}_{a}+{\left|u-u_{N}\right|}_{\sigma }+{\left\|p-p_{T}\right\|}_{M}\right\}\text{.}$$

A triangle inequality together with a Young inequality yields with a new constant C>0

(4.67) $${\left\|p-p_{N}\right\|}_{M}^{2}\leq {\left({\left\|p-p_{T}\right\|}_{M}+{\left\|p_{T}-p_{N}\right\|}_{M}\right)}^{2}\leq C\left\{{\left|u-u_{N}\right|}_{a}^{2}+{\left|u-u_{N}\right|}_{\sigma }^{2}+{\left\|p-p_{T}\right\|}_{M}^{2}\right\}\text{.}$$

We multiply this last equation with a constant and add it to (4.59). Choosing this constant sufficiently small to be able to absorb the terms ${\left|u-u_{N}\right|}_{a}^{2}$ and ${\left|u-u_{N}\right|}_{\sigma }^{2}$ from the right-hand side, we end up with

(4.68) $${\left|u-u_{N}\right|}_{a}^{2}+{\langle d-d_{N}\;;\;m-m_{N}\rangle }_{\Omega }+{\left|u-u_{N}\right|}_{\sigma }^{2}+{\left\|p-p_{N}\right\|}_{M}^{2}\leq C_{12}\left\{{\left|u_{T}^{⋆}-u\right|}_{a}^{2}+{\left\|m-m_{T}^{⋆}\right\|}_{\Omega }^{2}+{\left\|p-p_{T}\right\|}_{\overset{˜}{T}}^{2}+{\left|u_{T}^{⋆}-u\right|}_{\sigma }^{2}+{\left\|p-p_{T}\right\|}_{M}^{2}\right\}+c_{13}{\left\|d-d_{N}\right\|}_{\Omega }^{2}+c_{14}{\left\|\ell -\ell_{N}\right\|}_{\Omega }^{2}+C_{15}\left\{{\left\|\varepsilon^{1/2}\ell \right\|}_{L^{2}\left(\Omega \right)}^{2}-{\left\|\varepsilon^{1/2}\ell_{N}\right\|}_{L^{2}\left(\Omega \right)}^{2}\right\}\text{.}$$

***Step* 8:** Step 7 shows that for arbitrary $p_{T}\in M_{N}$, we have the best approximation result in the constrained space $\ker_{N}\;b$

(4.69) $${\left|u-u_{N}\right|}_{a}^{2}+{\langle d-d_{N}\;;\;m-m_{N}\rangle }_{\Omega }+{\left|u-u_{N}\right|}_{\sigma }^{2}+{\left\|p-p_{N}\right\|}_{M}^{2}\leq C_{12}\underset{u_{T}^{*}\in \ker_{N}\;b}{\inf}\left\{\overset{2}{\underset{a}{\left|\overset{⋆}{\underset{T}{u}}-u\right|}}+{\left\|m-m_{T}^{⋆}\right\|}_{\Omega }^{2}+{\left|u_{T}^{⋆}-u\right|}_{\sigma }^{2}\right\}+C_{12}\left\{{\left\|p-p_{T}\right\|}_{\overset{˜}{T}}^{2}+{\left\|p-p_{T}\right\|}_{M}^{2}\right\}+c_{13}{\left\|d-d_{N}\right\|}_{\Omega }^{2}+c_{14}{\left\|\ell -\ell_{N}\right\|}_{\Omega }^{2}+C_{15}\left\{{\left\|\varepsilon^{1/2}\ell \right\|}_{L^{2}\left(\Omega \right)}^{2}-{\left\|\varepsilon^{1/2}\ell_{N}\right\|}_{L^{2}\left(\Omega \right)}^{2}\right\}\text{.}$$

To finish the proof, we need to estimate

(4.70) $$\underset{u_{T}^{*}\in \ker_{N}\;b}{\inf}\left\{\overset{2}{\underset{a}{\left|\overset{⋆}{\underset{T}{u}}-u\right|}}+{\left\|m-m_{T}^{⋆}\right\|}_{\Omega }^{2}+{\left|u_{T}^{⋆}-u\right|}_{\sigma }^{2}\right\}\text{.}$$

Let $u_{T}=\left(u_{T},m_{T},\phi_{T}\right)\in X_{N}$ be arbitrary but fixed and u=(u,m,ϕ) be the exact solution of Problem 3.1(SPP). We now construct a correction $r_{N}=\left(r_{N},s_{N},\tau_{N}\right)\in X_{N}$ such that $u_{T}+r_{N}\in \ker_{N}\;b$. That is, we have to satisfy

(4.71) $$b\left(r_{N};q_{N}\right)=b\left(u-u_{T};q_{N}\right)\;\text{for all}\;q_{N}\in M_{N}\text{.}$$

The discrete inf–sup condition ensures solvability of (4.71), i.e., there exists a $r_{N}\in X_{N}$ such that

(4.72) $$\beta {\left\|r_{N}\right\|}_{X}\leq \underset{q_{N}\in M_{N}\setminus \left\{0\right\}}{\sup}\frac{b\left(r_{N};q_{N}\right)}{{\left\|q_{N}\right\|}_{M}}\leq \underset{q_{N}\in M_{N}\setminus \left\{0\right\}}{\sup}\frac{C_{b,2}{\left\|u-u_{T}\right\|}_{X}{\left\|q_{N}\right\|}_{M}}{{\left\|q_{N}\right\|}_{M}}=C_{b,2}{\left\|u-u_{T}\right\|}_{X}\text{,}$$

with the inf–sup constant $\beta =\min\left\{1,c_{1}^{V}\right\}$. This result and $u_{T}+r_{N}=\left(u_{T}+r_{N},m_{T}+s_{N},\phi_{T}+\tau_{N}\right)\in \ker_{N}\;b$ yields together with Lemmas 4.11 and 4.12

$$\underset{u_{T}^{⋆}\in \ker_{N}\;b}{\inf}\left\{\overset{2}{\underset{a}{\left|u-\overset{⋆}{\underset{T}{u}}\right|}}+{\left\|m-m_{T}^{⋆}\right\|}_{\Omega }^{2}+{\left|u-u_{T}^{⋆}\right|}_{\sigma }^{2}\right\}\leq {\left|u-\left(u_{T}+r_{N}\right)\right|}_{a}^{2}+{\left\|m-\left(m_{T}+s_{N}\right)\right\|}_{\Omega }^{2}+{\left|u-\left(u_{T}+r_{N}\right)\right|}_{\sigma }^{2}\leq 2\left\{{\left|u-u_{T}\right|}_{a}^{2}+{\left|r_{N}\right|}_{a}^{2}+{\left\|m-m_{T}\right\|}_{\Omega }^{2}+{\left\|s_{N}\right\|}_{\Omega }^{2}+{\left|u-u_{T}\right|}_{\sigma }^{2}+{\left|r_{N}\right|}_{\sigma }^{2}\right\}\leq 2\left\{2C_{a,X}^{2}{\left\|u-u_{T}\right\|}_{X}^{2}+2C_{a,X}^{2}{\left\|r_{N}\right\|}_{X}^{2}+{\left|u-u_{T}\right|}_{\sigma }^{2}+C_{\sigma }^{2}{\left\|r_{N}\right\|}_{X}^{2}\right\}\leq C\left\{{\left\|u-u_{T}\right\|}_{X}^{2}+{\left|u-u_{T}\right|}_{\sigma }^{2}\right\}\text{,}$$

where C>0 is appropriate. Plugging this into (4.69) leads us to

(4.73) $${\left|u-u_{N}\right|}_{a}^{2}+{\langle d-d_{N}\;;\;m-m_{N}\rangle }_{\Omega }+{\left|u-u_{N}\right|}_{\sigma }^{2}+{\left\|p-p_{N}\right\|}_{M}^{2}\leq C_{16}\left\{{\left\|u-u_{T}\right\|}_{X}^{2}+{\left|u-u_{T}\right|}_{\sigma }^{2}+{\left\|p-p_{T}\right\|}_{\overset{˜}{T}}^{2}+{\left\|p-p_{T}\right\|}_{M}^{2}+{\left\|\varepsilon^{1/2}\ell \right\|}_{L^{2}\left(\Omega \right)}^{2}-{\left\|\varepsilon^{1/2}\ell_{N}\right\|}_{L^{2}\left(\Omega \right)}^{2}\right\}+c_{13}{\left\|d-d_{N}\right\|}_{\Omega }^{2}+c_{14}{\left\|\ell -\ell_{N}\right\|}_{\Omega }^{2}\text{,}$$

which ends the proof. □

Proof of Theorem 4.9 ***Step* 1:** With the additional assumption (4.27) we absorb the term ${\left\|d-d_{N}\right\|}_{\Omega }^{2}$ on the right-hand side of (4.25) of Theorem 4.8 in the left-hand side:

(4.74) $${\left|u-u_{N}\right|}_{a}^{2}+{\left\|d-d_{N}\right\|}_{\Omega }^{2}+{\left|u-u_{N}\right|}_{\sigma }^{2}+{\left\|p-p_{N}\right\|}_{M}^{2}\leq C_{1}\left\{{\left\|u-u_{T}\right\|}_{X}^{2}+{\left|u-u_{T}\right|}_{\sigma }^{2}+{\left\|p-p_{T}\right\|}_{\overset{˜}{T}}^{2}+{\left\|p-p_{T}\right\|}_{M}^{2}+{\left\|\varepsilon^{1/2}\ell \right\|}_{\Omega }^{2}-{\left\|\varepsilon^{1/2}\ell_{N}\right\|}_{\Omega }^{2}\right\}+c_{2}{\left\|\ell -\ell_{N}\right\|}_{\Omega }^{2}\text{;}$$

here, $c_{2}>0$ is still arbitrary.

***Step* 2:** We claim that

(4.75) $${\left|u-u_{N}\right|}_{a}^{2}+{\left\|d-d_{N}\right\|}_{\Omega }^{2}+{\left\|\ell -\ell_{N}\right\|}_{\Omega }^{2}+{\left|u-u_{N}\right|}_{\sigma }^{2}+{\left\|p-p_{N}\right\|}_{M}^{2}\leq C_{2}\left\{{\left\|u-u_{T}\right\|}_{X}^{2}+{\left|u-u_{T}\right|}_{\sigma }^{2}+{\left\|p-p_{T}\right\|}_{\overset{˜}{T}}^{2}+{\left\|p-p_{T}\right\|}_{M}^{2}+{\left\|\varepsilon^{1/2}\ell \right\|}_{\Omega }^{2}-{\left\|\varepsilon^{1/2}\ell_{N}\right\|}_{\Omega }^{2}+{\left\|\ell -\Pi \ell \right\|}_{\Omega }^{2}\right\}\text{.}$$

Indeed, using the $L^{2}{\left(\Omega \right)}^{d}$-orthogonal projection, the Galerkin orthogonality (4.30) with $v_{N}=\left(0,\Pi \ell -\ell_{N},0\right)$, Lemma 4.12, and the Cauchy–Schwarz inequality, we get

(4.76) $${\left\|\Pi \ell -\ell_{N}\right\|}_{\Omega }^{2}={\langle \ell -\ell_{N}\;;\;\Pi \ell -\ell_{N}\rangle }_{\Omega }=-{\langle d-d_{N}\;;\;\Pi \ell -\ell_{N}\rangle }_{\Omega }-\sigma \left(u-u_{N};v_{N}\right)-{\langle \Pi \ell -\ell_{N}\;;\;\nabla \left(p-p_{N}\right)\rangle }_{\Omega }.\leq \left({\left\|d-d_{N}\right\|}_{\Omega }+C_{\sigma }{\left|u-u_{N}\right|}_{\sigma }+{\left\|\nabla \left(p-p_{N}\right)\right\|}_{\Omega }\right){\left\|\Pi \ell -\ell_{N}\right\|}_{\Omega }\text{.}$$

Cancelling the factor ${\left\|\Pi \ell -\ell_{N}\right\|}_{\Omega }$ on both sides and squaring the inequality gives

(4.77) $${\left\|\Pi \ell -\ell_{N}\right\|}_{\Omega }^{2}\leq 3C_{\sigma }^{2}\left\{{\left\|d-d_{N}\right\|}_{\Omega }^{2}+{\left|u-u_{N}\right|}_{\sigma }^{2}+{\left\|\nabla \left(p-p_{N}\right)\right\|}_{M}^{2}\right\}\text{.}$$

Using now the triangle inequality ${\left\|\ell -\ell_{N}\right\|}_{\Omega }^{2}\leq 2{\left\|\ell -\Pi \ell \right\|}_{\Omega }^{2}+2{\left\|\Pi \ell -\ell_{N}\right\|}_{\Omega }^{2}$ together with (4.74) yields (4.75).

***Step* 3:** In this last step, the claimed estimate (4.28) is proved. The following relation, valid for all positive constants C, was proven in [20, Lemma 2.32] (see also  [4]):

(4.78) $$C\left\{{\left\|\varepsilon^{1/2}\ell \right\|}_{L^{2}\left(\Omega \right)}^{2}-{\left\|\varepsilon^{1/2}\ell_{N}\right\|}_{L^{2}\left(\Omega \right)}^{2}\right\}\leq C^{2}\left\{{\left\|\varepsilon \right\|}_{L^{\infty }\left(\Omega \right)}{\left\|\varepsilon^{1/2}\ell \right\|}_{L^{2}\left(\Omega \right)}^{2}+{\left\|\varepsilon \right\|}_{L^{\infty }\left(\Omega \right)}{\left\|\varepsilon^{1/2}\ell_{N}\right\|}_{L^{2}\left(\Omega \right)}^{2}\right\}+\frac{1}{2}{\left\|\ell -\ell_{N}\right\|}_{L^{2}\left(\Omega \right)}^{2}\text{.}$$

Plugging this into (4.75) with $C=C_{2}$ and absorbing the term $\frac{1}{2}{\left\|\ell -\ell_{N}\right\|}_{L^{2}\left(\Omega \right)}^{2}$ gives

(4.79) $${\left|u-u_{N}\right|}_{a}^{2}+{\left\|d-d_{N}\right\|}_{\Omega }^{2}+{\left\|\ell -\ell_{N}\right\|}_{\Omega }^{2}+{\left|u-u_{N}\right|}_{\sigma }^{2}+{\left\|p-p_{N}\right\|}_{M}^{2}\leq 2C_{2}\left\{{\left\|u-u_{T}\right\|}_{X}^{2}+{\left|u-u_{T}\right|}_{\sigma }^{2}+{\left\|p-p_{T}\right\|}_{\overset{˜}{T}}^{2}+{\left\|p-p_{T}\right\|}_{M}^{2}+{\left\|\ell -\Pi \ell \right\|}_{\Omega }^{2}\right\}+2C_{2}^{2}{\left\|\varepsilon \right\|}_{L^{\infty }\left(\Omega \right)}\left\{{\left\|\varepsilon^{1/2}\ell \right\|}_{\Omega }^{2}+{\left\|\varepsilon^{1/2}\ell_{N}\right\|}_{\Omega }^{2}\right\}\text{.}$$

Finally, the term ${\left\|\varepsilon^{1/2}\ell_{N}\right\|}_{\Omega }^{2}$ can be estimated using (4.75) resulting in the claimed bound (4.28).

Remark 4.13 **Choice of Penalty Parameter ε**

The estimate  (4.29) is optimal with respect to the local mesh size h and suggests the choice $\varepsilon =O\left(h^{\alpha }\right)$ with α=1 in order to balance the upper estimate in (4.29). Numerical experiments (not shown here) reveal that the choice α∈(0,1) dominates the error in the sense that, for smooth exact solution (u,m), one observes numerically a convergence behavior $O\left(h^{\alpha }\right)$. In the experiment in Section  4.5, we choose the T-piecewise constant penalization function ε=h, where $h\in L^{\infty }\left(\Omega \right)$ is defined by $h{|}_{K}≔\mathrm{diam}\;K$.  ■

### 4.5 Numerical example

**Fig. 1:**
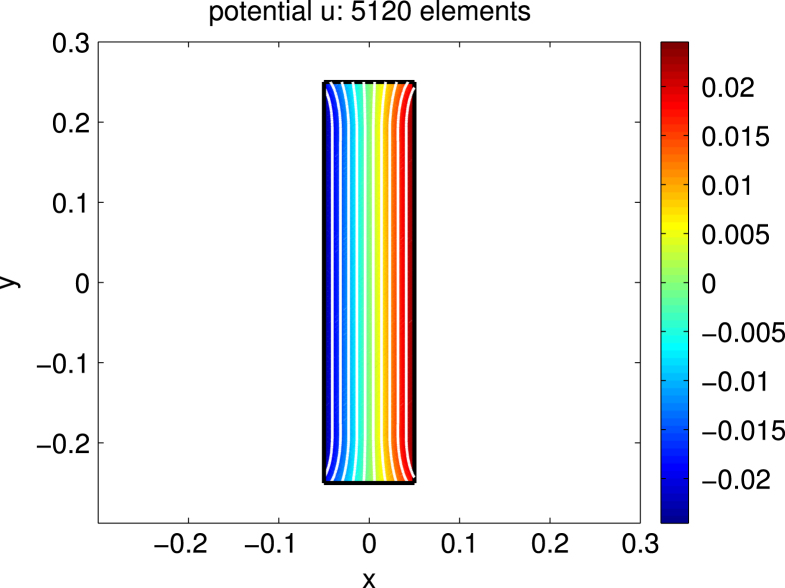
Potential $u_{N}$.

**Fig. 2:**
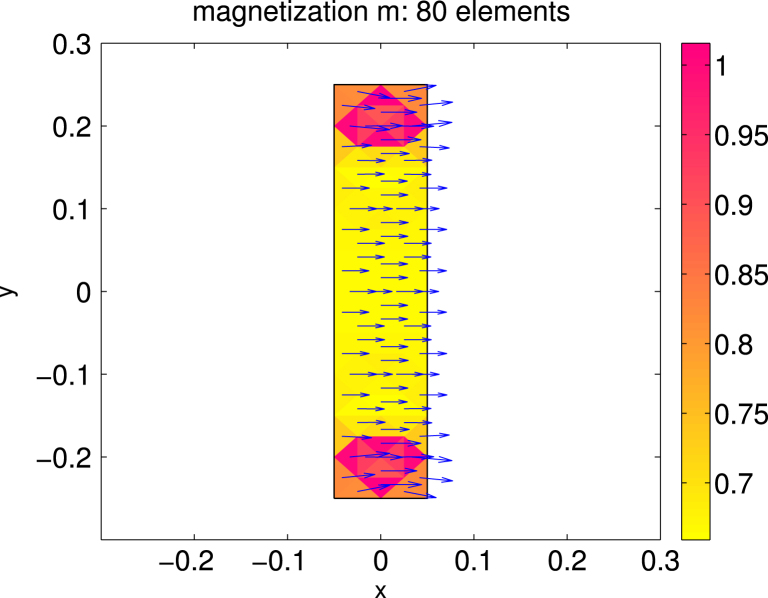
Magnetization $m_{N}$.

**Fig. 3:**
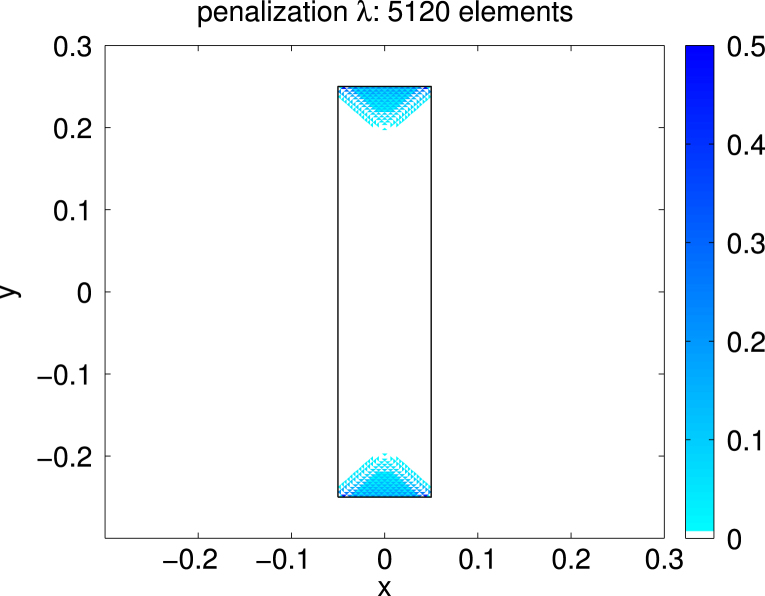
Penalization $\lambda_{N}$.

**Fig. 4:**
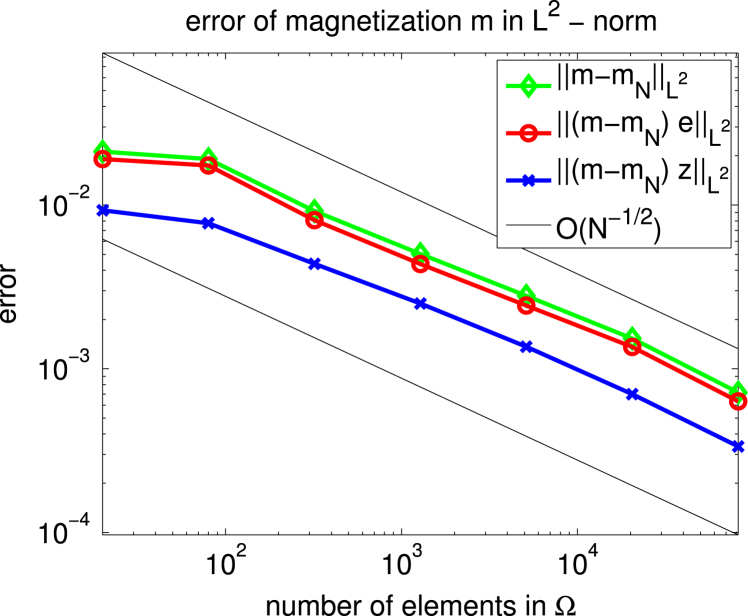
Convergence of m.

**Fig. 5:**
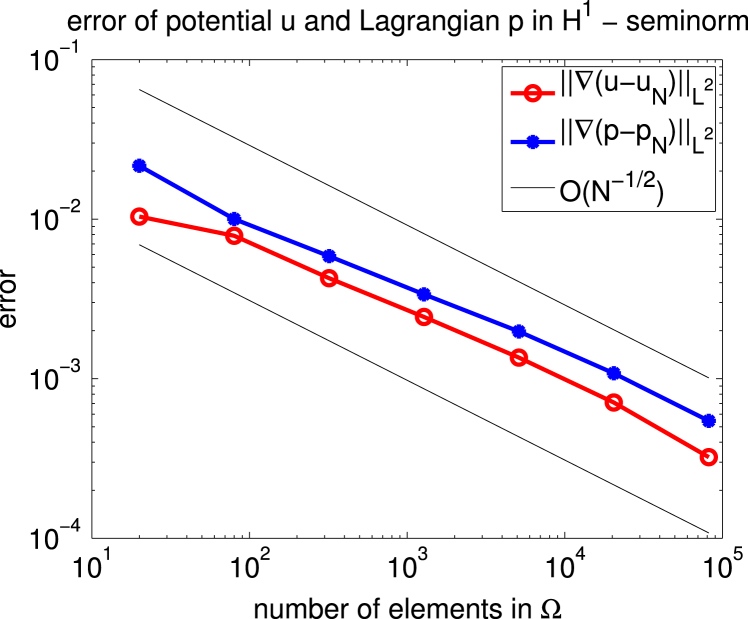
Convergence of u and p.

**Fig. 6:**
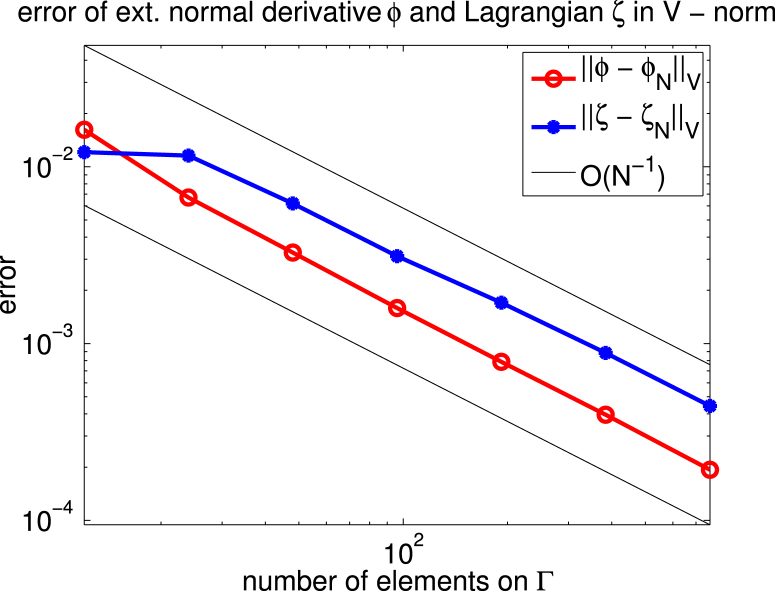
Convergence of ϕ and ζ.

For $\Omega =\left(-0.05,0.05\right)\times \left(-0.25,0.25\right)\subset R^{2}$ we consider the case of uniaxial materials discussed in Example 1.2 with easy axis e=[1,0] and correspondingly $z_{1}=z=\left[0,1\right]$. The exterior applied field f=[0.6,0] is constant and parallel to e. Up to scaling, this set of data coincides with an example already studied in [4]. Fig. 1 shows the isolines of the magnetic potential $u_{N}$ on the magnetic rod Ω whereas Fig. 2 presents the magnetization $m_{N}$ on a rather coarse mesh. Fig. 3 indicates the area of the rod Ω, where the penalization $\lambda_{N}$ is active. The convergence studies in Figs. 4–6 correspond to computations on a sequence of uniformly refined meshes $T_{\ell }$, $\ell =0,1,\ldots ,\ell_{max}-1$. The error is computed using a reference solution obtained on the finest mesh $T_{\ell_{max}}$. Fig. 4 presents the convergence ${\left\|\left(m-m_{N}\right)\cdot e\right\|}_{L^{2}\left(\Omega \right)}$ and ${\left\|\left(m-m_{N}\right)\cdot z\right\|}_{L^{2}\left(\Omega \right)}$ versus the number of elements in Ω. Although our *a priori* estimates do not provide control over ${\left\|\left(m-m_{N}\right)\cdot e\right\|}_{L^{2}\left(\Omega \right)}$, we observe good convergence. Fig. 5 shows the convergence of ${\left\|\nabla \left(u-u_{N}\right)\right\|}_{L^{2}\left(\Omega \right)}$ and ${\left\|\nabla \left(p-p_{N}\right)\right\|}_{L^{2}\left(\Omega \right)}$ versus the number of elements in Ω. Finally, Fig. 6 shows the performance for the errors $\phi -\phi_{N}$ and $\zeta -\zeta_{N}$. We measure the error in the norm ${\left\|\cdot \right\|}_{V}$ induced by the simple layer operator (see (2.7)) and plot the error versus the number of boundary elements.

## Appendix Proof of Lemma 2.2

Proof Proof of Lemma 2.2 • Item (i) is shown in Lemma A.1. • Items (ii) (i.e.,  (2.10)), (iii) (i.e.,  (2.11)), and (vi) (i.e.,  (2.14)) are shown in Lemma A.2. • Item (iv) (i.e., Eqs. (2.12) and (2.13)) is a direct consequence of  (2.11), see, e.g.,  [8–11]. • The assertion (v) of Lemma 2.2 follows from (2.13) and the fact that W1=0 and (1/2+K)1=0. □

Lemma A.1 *Let* $\Omega \subset R^{d}$ *be a bounded Lipschitz domain. Let* $u\in L_{loc}^{2}\left(\Omega^{\mathrm{ext}}\right)$ *satisfy*   (2.8)*,*   (2.9)*. Then* $u\in H^{1}\left(B_{R}\cap \Omega^{\mathrm{ext}}\right)$ *for every open ball* $B_{R}$ *with radius* R *such that* $\bar{\Omega }\subset B_{R}$ *. In particular,* $\gamma^{\mathrm{ext}}u\in H^{1/2}\left(\Gamma \right)$ *and* $\partial_{\nu }^{\mathrm{ext}}u\in H^{-1/2}\left(\Gamma \right)$ *are well-defined. Furthermore,*

(A.1) $${\langle \nabla u\;;\;\nabla \eta \rangle }_{L^{2}\left(\Omega^{\mathrm{ext}}\right)}=-{\langle \partial_{\nu }^{\mathrm{ext}}u\;;\;\eta \rangle }_{\Gamma }\;\text{for all}\;\eta \in \left\{v\in H^{1}\left(R^{d}\right)|\mathrm{supp}\left(v\right)\text{ compact}\right\}\text{.}$$

Proof We show $u\in H^{1}\left(B_{R}\cap \Omega^{\mathrm{ext}}\right)$. From this, the assertions about $\gamma^{\mathrm{ext}}u$ and $\partial_{\nu }^{\mathrm{ext}}u$ follow as well as (A.1) from an integration by parts  [31]. To see the assertion $u\in H^{1}\left(B_{R}\cap \Omega^{\mathrm{ext}}\right)$, it suffices to show $u\in L^{2}\left(B_{R}\cap \Omega^{\mathrm{ext}}\right)$. Since interior regularity for the Laplace operator implies $u\in C^{\infty }\left(\Omega^{\mathrm{ext}}\right)$, we only have to check the integrability of ${\left|u\right|}^{2}$ near Γ. This follows from $\nabla u\in L^{2}\left(\Omega^{\mathrm{ext}}\right)$ and standard arguments in the following way: Locally, the Lipschitz boundary Γ has the form $\left\{\left(x^{\prime },\varphi \left(x^{\prime }\right)\right)|x^{\prime }\in B^{\prime }\right\}$, where $B_{r}^{\prime }\subset R^{d-1}$ is a ball of radius r, and the cylinder $C_{2h}$ given by $C_{2h}≔\left\{\left(x^{\prime },y\right)|\varphi \left(x^{\prime }\right)<y<\varphi \left(x^{\prime }+2h\right),\;x^{\prime }\in B_{r}^{\prime }\right\}$ satisfies $C_{2h}\subset \Omega^{\mathrm{ext}}$. Set $\Gamma_{h}≔\left\{\left(x^{\prime },\varphi \left(x^{\prime }\right)+h\right)|x^{\prime }\in B_{r}^{\prime }\right\}\subset \Omega^{\mathrm{ext}}$ and note that by the smoothness of u we have ${\left\|u\right\|}_{L^{\infty }\left(\Gamma_{h}\right)}<\infty$. A 1D Sobolev embedding yields ${\left\|u\left(x^{\prime },\cdot \right)\right\|}_{L^{2}\left(\varphi \left(x^{\prime }\right),\varphi \left(x^{\prime }\right)+h\right)}≲\left\|u\left(x^{\prime },h\right)\right\|+{\left\|\nabla u\left(x^{\prime },\cdot \right)\right\|}_{L^{2}\left(\varphi \left(x^{\prime }\right),\varphi \left(x^{\prime }\right)+h\right)}$. An integration in $x^{\prime }$ yields ${\left\|u\right\|}_{L^{2}\left(C_{h}\right)}≲{\left\|\nabla u\right\|}_{L^{2}\left(C_{h}\right)}+{\left\|u\right\|}_{L^{\infty }\left(\Gamma_{h}\right)}$. Since a neighborhood of Γ is covered by finitely many cylinders of this form, the proof is complete. □

Lemma A.2 *Let* $\Omega \subset R^{d}$*,* d∈{2,3} *be a bounded Lipschitz domain and* $\Omega^{\mathrm{ext}}≔R^{d}\setminus \bar{\Omega }$ *. For* R>0*, let* $B_{R}\subset R^{d}$ *denote the ball with radius* R *centered at the origin. Then, any* u *satisfying*

(i) $-\Delta u=0\;\text{ in }\Omega^{\mathrm{ext}}$ *;*
(ii) $u{|}_{\Omega^{+}\cap B_{R}}\in H^{1}\left(\Omega^{\mathrm{ext}}\cap B_{R}\right)\;\forall R>0\;\text{sufficiently large}$ *;*
(iii) ${\left\|\nabla u\right\|}_{L^{2}\left(\Omega^{\mathrm{ext}}\right)}<\infty$ *(“finite energy”);*

*satisfies the radiation condition*   (2.10)*, the representation formula*   (2.11)*, and the energy representation formula*   (2.14)*.*

Proof The proof is broken up into several steps. In Step 2, we will show the representation formula (2.11); in Step 3, we prove the radiation condition (2.10); in Step 4, we ascertain the validity of the energy representation formula (2.14). **Step 1:** We claim that

(A.2) $$\underset{R\to \infty }{\lim}R^{1/2}{\left\|\nabla u\right\|}_{L^{2}\left(\partial B_{R}\right)}=0\text{.}$$

To see this, let R be sufficiently large. Using the multiplicative trace inequality and a standard scaling argument, we get

$${\left\|\nabla u\right\|}_{L^{2}\left(\partial B_{R}\right)}^{2}\leq C\left[R^{-1}{\left\|\nabla u\right\|}_{L^{2}\left(B_{2R}\setminus B_{R}\right)}^{2}+{\left\|\nabla u\right\|}_{L^{2}\left(B_{2R}\setminus B_{R}\right)}{\left|\nabla u\right|}_{H^{1}\left(B_{2R}\setminus B_{R}\right)}\right]\text{.}$$

Since the components of ∇u are harmonic functions on $B_{3R}\setminus B_{R/2}$ (for R sufficiently large), we get by the Caccioppoli inequality (see, e.g.,  [32, eqn. (5.3.12)]) the bound ${\left|\nabla u\right|}_{H^{1}\left(B_{2R}\setminus B_{R}\right)}\leq CR^{-1}{\left\|\nabla u\right\|}_{L^{2}\left(B_{3R}\setminus B_{R/2}\right)}$. We therefore conclude ${\left\|\nabla u\right\|}_{L^{2}\left(\partial B_{R}\right)}^{2}\leq CR^{-1}{\left\|\nabla u\right\|}_{L^{2}\left(B_{3R}\setminus B_{R/2}\right)}^{2}$. The assumption ${\left\|\nabla u\right\|}_{L^{2}\left(\Omega^{\mathrm{ext}}\right)}<\infty$ implies $\underset{R\to \infty }{\lim}{\left\|\nabla u\right\|}_{L^{2}\left(\Omega^{\mathrm{ext}}\setminus B_{R}\right)}=0$, which in turn implies (A.2). **Step 2:** We claim the existence of a constant $u_{\infty }\in R$ such that for $x\in \Omega^{\mathrm{ext}}$ we have the following representation formula

(A.3) $$u\left(x\right)=\int_{\partial \Omega }G\left(x,y\right)\partial_{\nu }^{\mathrm{ext}}u\;ds_{y}-\int_{\partial \Omega }\partial_{\nu \left(y\right)}^{\mathrm{ext}}G\left(x,y\right)\gamma^{\mathrm{ext}}u\;ds_{y}+u_{\infty }\text{.}$$

To see the representation (A.3), fix $x\in \Omega^{\mathrm{ext}}$, assume $x\in B_{R}$, and compute with the representation formula for the “annulus” $B_{R}\setminus \Omega$:

$$u\left(x\right)=\int_{\partial \Omega }G\left(x,y\right)\partial_{\nu }^{\mathrm{ext}}u\;ds_{y}-\int_{\partial \Omega }\partial_{\nu \left(y\right)}^{\mathrm{ext}}G\left(x,y\right)\gamma^{\mathrm{ext}}u\;ds_{y}-\int_{\partial B_{R}}G\left(x,y\right)\partial_{\nu \left(y\right)}u\;ds_{y}+\int_{\partial B_{R}}\partial_{\nu \left(y\right)}G\left(x,y\right)u\;ds_{y}\text{,}$$

where $\partial_{\nu }$ denote the (outer) normal derivative on $\partial B_{R}$. Let ${\bar{u}}_{R}=\frac{1}{\left|\partial B_{R}\right|}\int_{\partial B_{R}}u\;ds_{y}$ be the average of u on $\partial B_{R}$. Since $x\in B_{R}$, we have by the jump relations satisfied by the double layer potential $\int_{\partial B_{R}}\partial_{\nu \left(y\right)}G\left(x,y\right)\;ds_{y}=-1$. Hence, we can compute

$$\int_{\partial B_{R}}\partial_{\nu \left(y\right)}G\left(x,y\right)u\;ds_{y}=\int_{\partial B_{R}}\partial_{\nu \left(y\right)}G\left(x,y\right)\left(u-{\bar{u}}_{R}\right)\;ds_{y}-{\bar{u}}_{R}$$

so that we obtain the representation

(A.4) $$u\left(x\right)=\int_{\partial \Omega }G\left(x,y\right)\partial_{\nu }^{\mathrm{ext}}u\;ds_{y}-\int_{\partial \Omega }\partial_{\nu \left(y\right)}^{\mathrm{ext}}G\left(x,y\right)\gamma^{\mathrm{ext}}u\;ds_{y}-\int_{\partial B_{R}}G\left(x,y\right)\partial_{\nu \left(y\right)}u\;ds_{y}+\int_{\partial B_{R}}\partial_{\nu \left(y\right)}G\left(x,y\right)\left(u-{\bar{u}}_{R}\right)\;ds_{y}-{\bar{u}}_{R}\text{.}$$

Let us first consider the case d=3. By the decay properties of G and $\partial_{\nu \left(y\right)}G$:

(A.5) $$\left|\int_{\partial B_{R}}G\left(x,y\right)\partial_{\nu \left(y\right)}u\;ds_{y}\right|\leq C\left(x\right)R^{-\left(d-2\right)}R^{\left(d-1\right)/2}{\left\|\nabla u\right\|}_{L^{2}\left(\partial B_{R}\right)}\leq C\left(x\right)R^{-\left(d-2\right)}R^{d/2-1}R^{1/2}{\left\|\nabla u\right\|}_{L^{2}\left(\partial B_{R}\right)}\text{,}$$

(A.6) $$\left|\int_{\partial B_{R}}\partial_{\nu \left(y\right)}G\left(x,y\right)\left(u-{\bar{u}}_{R}\right)\;ds_{y}\right|\leq C\left(x\right)R^{-\left(d-1\right)}R^{\left(d-1\right)/2}{\left\|u-{\bar{u}}_{R}\right\|}_{L^{2}\left(\partial B_{R}\right)}\leq C\left(x\right)R^{-\left(d-1\right)}R^{\left(d-1\right)/2}R{\left\|\nabla u\right\|}_{L^{2}\left(\partial B_{R}\right)}\text{;}$$

 in view of (A.2), we conclude that, as R→∞, the third and the fourth integral in (A.4) tend to zero. The first two integrals are (for fixed x) constant as is the left-hand side u(x). This shows that $\lim_{R\to \infty }{\bar{u}}_{R}$ exists:

$$u_{\infty }≔-\underset{R\to \infty }{\lim}{\bar{u}}_{R}=u\left(x\right)-\int_{\partial \Omega }G\left(x,y\right)\partial_{\nu }^{\mathrm{ext}}u\;ds_{y}+\int_{\partial \Omega }\partial_{\nu \left(y\right)}^{\mathrm{ext}}G\left(x,y\right)\gamma^{\mathrm{ext}}u\;ds_{y}\text{.}$$

This is the desired representation formula. We now consider the case d=2, which requires a more delicate reasoning due to the logarithmic growth of the fundamental solution G. We proceed by using pointwise estimates for ∇u(x). Differentiating (A.4) yields

$$\nabla_{x}u\left(x\right)=\nabla_{x}\int_{\partial \Omega }G\left(x,y\right)\partial_{\nu }^{\mathrm{ext}}u\;ds_{y}-\nabla_{x}\int_{\partial \Omega }\partial_{\nu \left(y\right)}^{\mathrm{ext}}G\left(x,y\right)\gamma^{\mathrm{ext}}u\;ds_{y}-\int_{\partial B_{R}}\nabla_{x}G\left(x,y\right)\partial_{\nu \left(y\right)}u\;ds_{y}+\int_{\partial B_{R}}\nabla_{x}\partial_{\nu \left(y\right)}G\left(x,y\right)\left(u-{\bar{u}}_{R}\right)\;ds_{y}\text{.}$$

The explicit formula for G yields for a C>0 that depends on $\gamma^{\mathrm{ext}}u$, $\partial_{\nu }^{\mathrm{ext}}u$, and ∂Ω

$$\left|\nabla_{x}\int_{\partial \Omega }G\left(x,y\right)\partial_{\nu }^{\mathrm{ext}}u\;ds_{y}\right|\leq C{\left|x\right|}^{-\left(d-1\right)}\text{,}\;\left|\nabla_{x}\int_{\partial \Omega }\partial_{\nu \left(y\right)}^{\mathrm{ext}}G\left(x,y\right)\gamma^{\mathrm{ext}}u\;ds_{y}\right|\leq C{\left|x\right|}^{-d}\text{.}$$

Furthermore, we get for fixed x with the Cauchy–Schwarz inequality and (A.2)

$$\left|\nabla_{x}\int_{\partial B_{R}}\partial_{\nu \left(y\right)}G\left(x,y\right)\left(u-{\bar{u}}_{R}\right)\;ds_{y}\right|\leq {\left\|\nabla_{x}\nabla_{y}G\left(x,\cdot \right)\right\|}_{L^{2}\left(\partial B_{R}\right)}{\left\|u-{\bar{u}}_{R}\right\|}_{L^{2}\left(\partial B_{R}\right)}\leq C\left(x\right)R^{-d}R^{\left(d-1\right)/2}R{\left\|\nabla u\right\|}_{L^{2}\left(\partial B_{R}\right)}\to 0\;\text{ as }R\to \infty ,\left|\nabla_{x}\int_{\partial B_{R}}G\left(x,y\right)\partial_{\nu \left(y\right)}u\;ds_{y}\right|\leq {\left\|\nabla_{x}G\left(x,\cdot \right)\right\|}_{L^{2}\left(\partial B_{R}\right)}{\left\|\nabla u\right\|}_{L^{2}\left(\partial B_{R}\right)}\leq C\left(x\right)R^{-\left(d-1\right)}R^{\left(d-1\right)/2}{\left\|\nabla u\right\|}_{L^{2}\left(\partial B_{R}\right)}\to 0\;\text{ as }R\to \infty \text{.}$$

The above developments show two things, namely, a representation formula for ∇u and an estimate:

(A.7) $$\nabla u\left(x\right)=\nabla_{x}\int_{\partial \Omega }G\left(x,y\right)\partial_{\nu }^{\mathrm{ext}}u\;ds_{y}-\nabla_{x}\int_{\partial \Omega }\partial_{\nu \left(y\right)}^{\mathrm{ext}}G\left(x,y\right)\gamma^{\mathrm{ext}}u\;ds_{y}\text{,}$$

(A.8) $$\left|\nabla u\left(x\right)\right|\leq C_{u}{\left|x\right|}^{-\left(d-1\right)}\;\text{ as }x\to \infty \text{.}$$

 The representation of the gradient (A.7) and an expansion of G yield for large x the asymptotic expression

$$\nabla u\left(x\right)=c\frac{x}{{\left|x\right|}^{2}}\int_{\partial \Omega }\partial_{\nu }^{\mathrm{ext}}u\;ds_{y}+O\left({\left|x\right|}^{-2}\right)\;\text{ as }\left|x\right|\to \infty \text{,}$$

where c≠0. Since we assume that $\nabla u\in L^{2}\left(\Omega^{\mathrm{ext}}\right)$, we conclude

(A.9) $$\int_{\partial \Omega }\partial_{\nu }^{\mathrm{ext}}u\;ds_{y}=0\text{.}$$

This implies that we can sharpen the estimate (A.8) to

(A.10) $$\left|\nabla u\left(x\right)\right|\leq C{\left|x\right|}^{-d}\text{,}\;\left|x\right|\to \infty \text{.}$$

This sharper bound can be fed back into (A.4): the third and the fourth integral can now be estimated by

$$\left|\int_{\partial B_{R}}G\left(x,y\right)\partial_{\nu \left(y\right)}u\;ds_{y}\right|\leq C\left(x\right)\ln R\;R^{\left(d-1\right)/2}{\left\|\nabla u\right\|}_{L^{2}\left(\partial B_{R}\right)},\leq C\left(x\right)\ln R\;R^{d-1}R^{-d},\left|\int_{\partial B_{R}}\partial_{\nu \left(y\right)}G\left(x,y\right)\left(u-{\bar{u}}_{R}\right)\;ds_{y}\right|\leq C\left(x\right)R^{-\left(d-1\right)}R^{\left(d-1\right)/2}{\left\|u-{\bar{u}}_{R}\right\|}_{L^{2}\left(\partial B_{R}\right)}\leq C\left(x\right)R^{-\left(d-1\right)}R^{d-1}RR^{-d}\text{.}$$

These two terms tend to zero as R→∞. Therefore, as in the case d=3, we obtain that $\lim_{R\to \infty }{\bar{u}}_{R}$ exists and conclude the argument in this case in exactly the same manner as in the case d=3. **Step 3:** We show $u=u_{\infty }+O\left(1/r\right)$. For the case d=3, this follows directly from the representation formula (A.3) and the decay properties of the potentials. For the case d=2, it follows from the representation formula (A.3) and the addition properties $\int_{\partial \Omega }\partial_{\nu }^{\mathrm{ext}}u\;ds_{y}=0$, which we proved in (A.9). **Step 4:** We show (2.14) using (A.1) and Lebesgue Dominated Convergence. Fix $R_{0}$ such that $\bar{\Omega }\subset B_{R_{0}/2}$. Define annuli $\Omega_{j}≔B_{2^{j}R_{0}}\setminus B_{2^{j-2}R_{0}}$ for j∈N and set $\Omega_{0}≔B_{R_{0}}$. Let ${\left(\varphi_{j}\right)}_{j\in N_{0}}\subset C_{0}^{\infty }\left(R^{d}\right)$ be a partition of unity on $R^{d}$ subordinate to the covering ${\left(\Omega_{j}\right)}_{j\in N_{0}}$. Note that $\varphi_{0}\equiv 1$ on ∂Ω and that $\varphi_{j}{|}_{\partial \Omega }=0$ for j≥1. We assume that additionally $\left|\nabla \varphi_{j}\left(x\right)\right|\leq C/\left|x\right|$ for $\left|x\right|\geq R_{0}$ and ${\left\|\varphi_{j}\right\|}_{L^{\infty }\left(R^{d}\right)}\leq C$. For each j, we have the pointwise estimate

$$\left|\nabla u\cdot \nabla \left(\varphi_{j}\left(u-u_{\infty }\right)\right)\right|≲\left|\nabla u\right|\left[\frac{\left|u-u_{\infty }\right|}{\left|x\right|}+\left|\nabla u\right|\right]≲{\left|\nabla u\right|}^{2}+\frac{1}{{\left|x\right|}^{4}}\text{,}$$

where we exploited our assumptions on $\nabla \varphi_{j}$ and the radiation condition (2.10). By the finite overlap properties of the partition of unity ${\left(\varphi_{j}\right)}_{j\in N}$ (each $x\in R^{d}$ is contained in the support of at most two functions $\varphi_{j}$), we obtain the pointwise estimate

$$\overset{\infty }{\underset{j=0}{\sum }}\left|\nabla u\cdot \nabla \left(\varphi_{j}\left(u-u_{\infty }\right)\right)\right|≲{\left|\nabla u\right|}^{2}+\frac{1}{{\left|x\right|}^{4}}\text{.}$$

This function is in $L^{1}\left(R^{d}\cap \Omega^{\mathrm{ext}}\right)$ for d∈{2,3}. Therefore, the Lebesgue Dominated Convergence theorem gives us

$${\left\|\nabla u\right\|}_{L^{2}\left(\Omega^{\mathrm{ext}}\right)}^{2}=\int_{R^{d}\cap \Omega^{\mathrm{ext}}}\nabla u\cdot \nabla \left(u-u_{\infty }\right)=\int_{R^{d}\cap \Omega^{\mathrm{ext}}}\overset{\infty }{\underset{j=0}{\sum }}\nabla u\cdot \nabla \left(\varphi_{j}\left(u-u_{\infty }\right)\right)=\overset{\infty }{\underset{j=0}{\sum }}\int_{R^{d}\cap \Omega^{\mathrm{ext}}}\nabla u\cdot \nabla \left(\varphi_{j}\left(u-u_{\infty }\right)\right)\overset{\text{(A.1)}}{=}\overset{\infty }{\underset{j=0}{\sum }}-\int_{\partial \Omega }\partial_{\nu }^{\mathrm{ext}}u\varphi_{j}\left(u-u_{\infty }\right)\;ds_{y}=-\int_{\partial \Omega }\partial_{\nu }^{\mathrm{ext}}u\left(u-u_{\infty }\right)\;ds_{y}\overset{\text{ Lemma 2.2, (v)}}{=}-\int_{\partial \Omega }\partial_{\nu }^{\mathrm{ext}}uu\;ds_{y}\text{,}$$

which is the desired formula (2.14).
